# Ecological momentary assessment of meal context and food types contributing to salt intake at meals

**DOI:** 10.1186/s12966-025-01780-1

**Published:** 2025-06-28

**Authors:** Nana Shinozaki, Kentaro Murakami, Shizuko Masayasu, Satoshi Sasaki

**Affiliations:** 1 Department of Social and Preventive Epidemiology, School of Public Health, The University of Tokyo, 7-3-1 Hongo, Bunkyo-ku, Tokyo, 113-0033 Japan; 2 Ikurien-naka, 3799-6 Sugaya, Naka-shi, Ibaraki, 311–0105 Japan

**Keywords:** Sodium, Eating behavior, Meal context, Ecological momentary assessment, Dietary record

## Abstract

**Background:**

Salt (sodium) intake can vary across meals depending on the meal context and food types, including what and how much was consumed and where, when, and with whom it was consumed. However, their dynamic associations remain unclear. This study examined how meal context and food types are associated with salt intake at meals.

**Methods:**

This cross-sectional analysis used data from 2757 adults aged 18–79 years. Ecological momentary assessment was conducted using eight-day dietary records to obtain information on meal context, food types, and salt intake.

**Results:**

Multilevel linear regression analysis of 63,239 meals showed a higher absolute salt intake (g/meal) at lunch (*β*: 0.47, 95% CI: 0.44, 0.51) and dinner (*β*: 0.84, 95% CI: 0.80, 0.88) than at breakfast. In addition, salt intake from meals eaten on non-working or non-school days (*β*: 0.10, 95% CI: 0.06, 0.13), in restaurants (*β*: 0.40, 95% CI: 0.34, 0.45), and with one other person (*β*: 0.08, 95% CI: 0.05, 0.12) was higher than that from meals eaten on working or school days, at home, and alone, respectively. Regarding food types, salt intake was significantly higher in meals containing staple foods (e.g., rice and bread), especially noodles (*β*: 2.29, 95% CI: 2.23, 2.36), as well as soup (*β*: 1.06, 95% CI: 1.03, 1.09), pickles (*β*: 0.72, 95% CI: 0.68, 0.75), reduced-salt seasonings (*β*: 0.35, 95% CI: 0.23, 0.47), herbs and spices (*β*: 0.13, 95% CI: 0.10, 0.16), citrus juice and vinegar (*β*: 0.30, 95% CI: 0.26, 0.34), moderately processed meat and seafood (*β*: 0.59, 95% CI: 0.56, 0.62), highly processed meat and seafood (*β*: 0.58, 95% CI: 0.55, 0.61), and alcoholic beverages (*β*: 0.36, 95% CI: 0.32, 0.41) than in meals without these foods. Consumption of salt-based seasonings and vegetables was positively associated with salt intake, whereas consuming fruit was associated with lower salt intake (*β*: -0.12, 95% CI: -0.15, -0.09). Using salt intake density (g/100 kcal) per meal instead of absolute salt intake showed generally similar associations.

**Conclusions:**

Meal context and food types were associated with salt intake. These findings would be useful for developing practical strategies for reducing salt intake.

**Supplementary Information:**

The online version contains supplementary material available at 10.1186/s12966-025-01780-1.

## Background

The direct link between sodium intake and the development of hypertension and cardiovascular diseases is widely recognized [1]. The World Health Organization (WHO) recommends that adults limit their sodium intake to less than 5 g/day of salt [1]. However, the average global sodium intake of adults is 10.8 g/day of salt, more than double the recommended amount [1]. Japan is known for its particularly high sodium intake, largely from seasonings [2], with an average salt intake of 14.0 g/day in males and 11.8 g/day in females, estimated based on 24-hour urinary sodium excretion [3].

Many countries have implemented diverse sodium intake reduction campaigns targeting the general population [1, 4–9], with nutrition education aimed at promoting individual behavior change being one of the most common strategies [10–12]. These efforts often emphasize specific behaviors related to meal context (e.g., when, where, and with whom meals are consumed) and food consumption (e.g., what and how much is eaten), such as avoiding eating out and reducing processed food consumption [13–19]. Previous studies have suggested that both meal context and food types may be associated with sodium intake [20–31]. For example, sodium intake tends to be higher when eating out [21, 25], at lunch or dinner [22–24], on weekends [26, 27], when eating alone [28], and among individuals with higher consumption of alcohol [29] and vegetables [30, 31]. However, since these associations have generally been examined separately, it remains unclear whether they are observed when considered simultaneously and how strongly each factor is associated with sodium intake. Moreover, most previous studies have focused on between-individual differences in sodium intake, such as differences based on eating-out frequency [21, 25], while little is known about the meal context and food types linked to within-person variation [22, 28]. Because meal context and food types can vary from meal to meal within the same individual [22, 28, 32], identifying the relative contributions of these variables to within-person variations in sodium intake is important for developing more targeted and effective sodium reduction strategies.

Ecological momentary assessment (EMA), which involves repeated collection of information regarding an individual’s current behavior and experiences over time in real-time and natural settings, can be useful in this regard [33–36]. This method collects data each time a specific event occurs (e.g., eating episodes) or at fixed intervals (e.g., every two hours) using various tools, such as written diaries, telephones, and electronic journals, with or without prompts for participants to complete the assessment [33]. EMA helps reduce recall bias and maximizes ecological validity (i.e., generalizability to daily life and natural settings) [33–36], and has been used to identify various contextual factors related to the nutritional quality of meals [22, 37–40]. Thus, meal context and food types that contribute to variation in sodium intake across meals can be effectively clarified through EMA of dietary data repeatedly recorded in real-world settings. However, to the best of our knowledge, no study has been conducted to comprehensively examine the associations between salt intake and contextual factors and food types to date.

Therefore, the aim of this study was to examine the relationships between sodium intake at each meal and meal context and food types among Japanese adults aged 18–79 years, using event-based EMA. We hypothesized that meal context and food types are associated with sodium intake from meals. The findings of this study will be important because they can serve as a basis for the development and implementation of future interventions, policies, and strategies for effective reduction of salt intake.

## Methods

### Data source

#### Study procedure

This cross-sectional study was conducted using data from MINistry of health, labour and welfare-sponsored NAtionwide study on Dietary intake Evaluation (MINNADE) survey [41]. The survey included two rounds of data collection conducted over one year (first round: November 2016 to September 2017; second round: October 2017 to September 2018). The target population included healthy, community-dwelling Japanese volunteers aged 1–79 years living in 32 of the 47 prefectures in Japan. A total of 441 research dietitians recruited volunteers using snowball sampling and collected relevant data. Considering feasibility and the availability of human and financial resources, we set a target of recruiting 256 individuals (128 males and 128 females) from each of the following nine age groups in the first data collection round: 1–6, 7–13, 14–19, 20–29, 30–39, 40–49, 50–59, 60–69, and 70–79 years (total *n =* 2304). Based on the dropout rates in each sex–age group in the first round, the target for the second round was set at 110–119 participants per group (*n* = 2051 in total), resulting in a total target sample size of 4355 participants.

The primary inclusion criterion was being a free-living individual willing to participate in the survey. Dietitians, individuals living with a dietitian, those working with a research dietitian, those who had received dietary counseling from a doctor or dietitian, those undergoing insulin or dialysis treatment, and pregnant or lactating women (at the beginning of the survey) were excluded from the study. Only one person per household was allowed to participate. As the snowball sampling method was used for participant recruitment, the number of individuals contacted was not recorded, making it impossible to calculate the response rate.

At the start of the survey, body weight (to the nearest 0.1 kg) and height (to the nearest 0.1 cm) were measured with the participant barefoot and in light clothing. The measurements were taken by either a family member or a research dietitian using standardized procedures. Participant characteristics were self-reported using paper questionnaires.

### *Ecological momentary assessment of food intake and meal context*

An event-based EMA was conducted to assess meal context and food consumption using paper-based dietary records (DRs). Participants were instructed to record the meal context and all foods and beverages consumed at the time of each eating occasion without receiving any prompts [33]. Details of the recording procedure are described below and have been reported previously [41]. DRs were collected for eight non-consecutive days, comprising two days of records for each of the four seasons. Each pair of recording days per season included two weekdays for half of the participants and one weekday plus one weekend day for the other half. This approach was used to allow for the collection of approximate overall dietary data in a 3:1 ratio (actual ratio, 5:2) of weekdays to weekends while maintaining feasibility and simplicity.

**Fig. 1:**
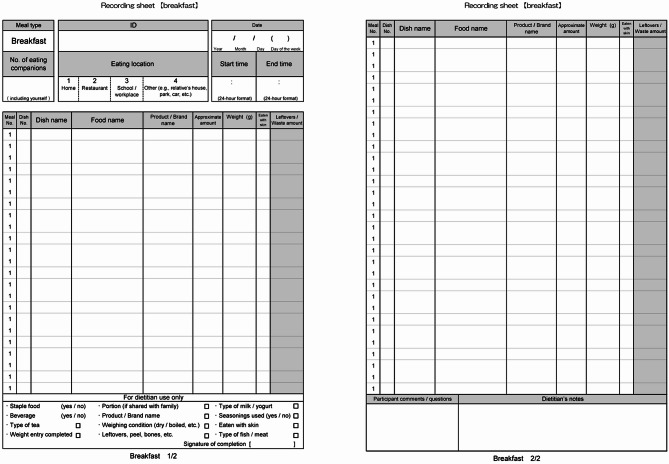
Example of a dietary recording sheet for breakfast

The research dietitians provided the participants with DR sheets along with verbal and written instructions on how to complete them. The DR sheets included sections for breakfast, lunch, dinner, and snacks (Fig. 1). For each meal, the following information was documented: (1) number of eating companions, (2) eating location, (3) the start and end times of eating, (4) names of dishes and foods, including ingredients, (5) product or brand names, and (6) measured weights or estimated amounts of foods and beverages consumed. The participants were requested to weigh foods and beverages whenever possible using a digital scale (KS-812WT; Tanita, Tokyo, Japan) that measures up to 2 kg in 1 g increments. If weighing was not feasible, the participants were asked to provide detailed information about the food, including the names of products, brands, restaurants, and estimated amounts consumed or left over. Participants were asked to record the names of all seasonings added to foods, including those added at the table, but were not required to measure their weights. This approach aimed to minimize participant burden and increase the feasibility of completing the DR. On each recording day, participants indicated whether that day was (a) a working or school day, (b) a non-working or non-school day, or (c) another type of schedule (e.g., retirees).

The recording sheets were collected by the research dietitians within a few days of each recording day, usually on the following day. The dietitians reviewed the records for completeness using a checklist provided on the DR sheet (Fig. 1), confirming all necessary details to identify food items and amounts consumed, such as the weight of foods and seasonings used. If any information was missing or unclear, they contacted participants in person, by phone, or by email as needed and added the necessary details to the records. In addition, they assigned each food item a food code from the seventh revised edition of the Standard Tables of Food Composition in Japan (STFCJ) [42]. For foods with no weight data recorded or with only approximate amounts, dietitians estimated the consumed ingredients and their weights as accurately as possible using various sources, such as standard portion sizes [43–45], the manufacturer’s website, and cookbooks. Other research dietitians at the survey’s central office verified all food codes and weights. After excluding dietary supplements, the eighth revised edition of the STFCJ [46], the latest version published after the MINNADE survey, was used to calculate the daily energy and sodium intakes of each participant based on the weight and nutrient content of the consumed food items.

### Data handling

#### Analytical sample

**Fig. 2:**
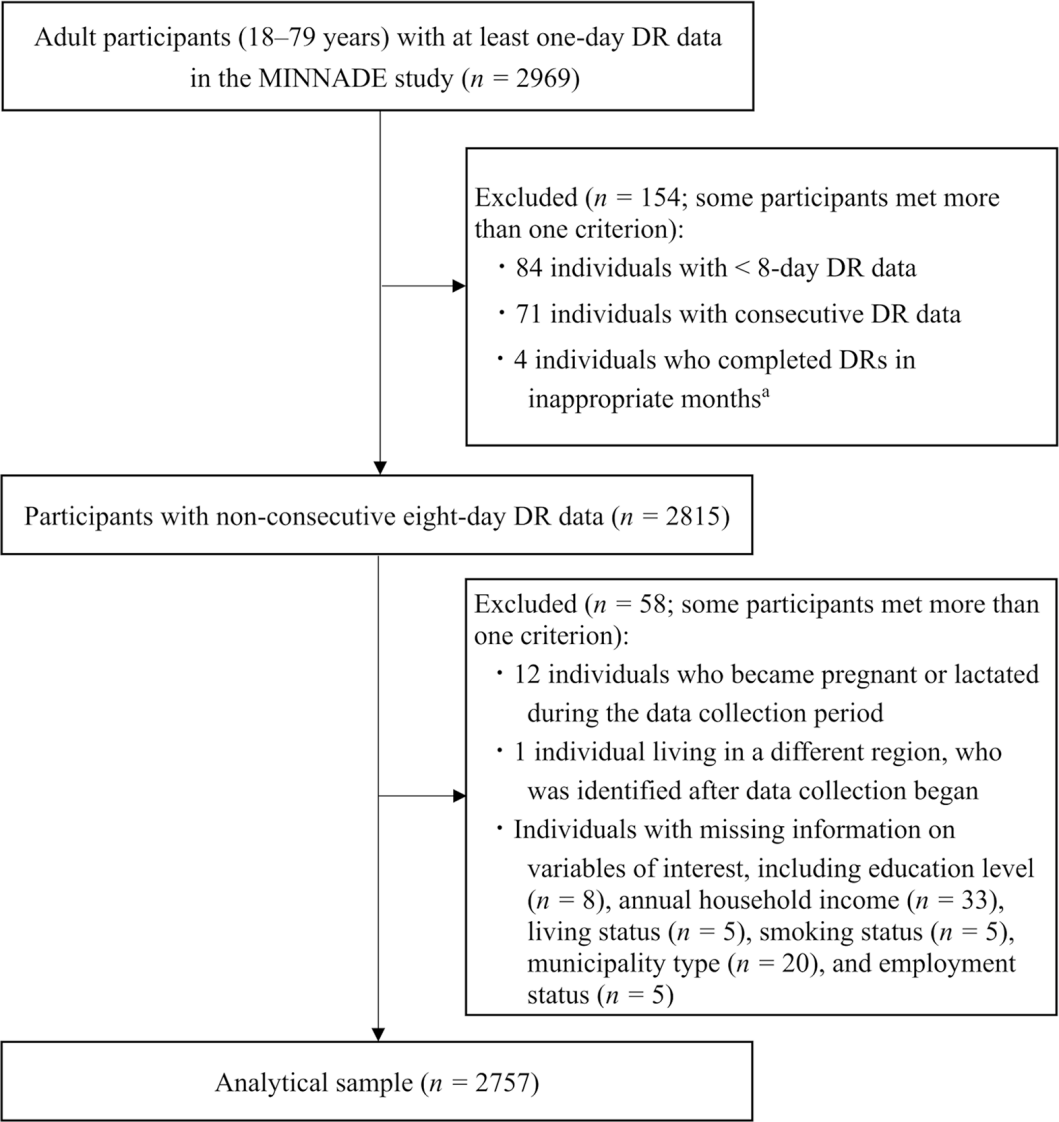
Flowchart of the participant selection process. DR, dietary record; MINNADE, MINistry of health, labour and welfare-sponsored Nationwide study on Dietary intake Evaluation. ^a^Appropriate months for each season were as follows: October, November, and December for fall; January, February, and March for winter; April, May, and June for spring; and July, August, and September for summer

A total of 4299 individuals aged 1–79 years participated in the MINNADE survey (first round: *n* = 2263; second round: *n* = 2036). Among them, 2969 adults aged 18–79 years completed at least one day of DRs (Fig. 2). We excluded 84 participants who completed DRs for fewer than eight days, 71 participants with consecutive DR data, four who did not complete the DRs during the designated months (October–December for fall; January–March for winter; April–June for spring; and July–September for summer), 12 participants who became pregnant or lactated during data collection, one participant living in a different region, who was identified after data collection began, and participants with missing information on the following variables of interest: education level (*n =* 8), annual household income (*n =* 33), living status (*n =* 5), smoking status (*n =* 5), municipality type (*n =* 20), and employment status (*n =* 5). As some participants met more than one criterion, 2757 participants aged 18–79 years were included in the final analysis. This study was reported in accordance with the guidelines in the adapted Strengthening the Reporting of Observational Studies in Epidemiology (STROBE) Checklist for Reporting EMA Studies [47] and the STROBE-nut reporting guidelines [48].

### *Analyzed meals*

In this study, meal types (breakfast, lunch, dinner, and snacks) were classified based on the section of the DR sheet in which the meal was recorded. In the present sample, snacks contributed only 2.5% of total sodium intake per person on average (standard deviation [SD]: 2.8%). Given their minimal contribution to sodium intake and the substantial differences in food types and quantities compared to main meals [24, 49], snacks were excluded from the analysis. After excluding snacks (*n* = 17,906), 64,588 meals were identified in the DRs of 2757 participants. Thereafter, we excluded 1328 meals that contained only beverages and 21 meals with missing information on eating location or companions. Therefore, 63,239 meals were included in the analysis. Energy and sodium intake from each meal were estimated using the eighth revised edition of the STFCJ [46]. Sodium intake was converted into absolute salt intake using the following formula: 1 g of sodium chloride equivalent = 58.5/23 × sodium (g) [50]. Additionally, salt intake density (g/100 kcal per meal) was calculated for each meal.

### *Meal context*

Meal context variables were generated from the information recorded on the DR sheet for each meal. Meals were classified according to seasons based on the month they were recorded: spring (April–June), summer (July–September), fall (October–December), and winter (January–March). For the day type variable, the “another type of schedule” category was combined with “non-working or non-school days” due to its low frequency, resulting in two categories: (1) working or school day and (2) non-working or non-school day. Eating location, selected from home, restaurant, school or workplace, or other places (e.g., cars and parks), was recategorized into three groups (home, restaurant, or other places) due to the unbalanced distribution of the data. Similarly, the number of eating companions was reclassified as eating alone, with one other person, or with two or more people.

### *Food type*

Food type variables, which represent the consumption of specific food items that may contribute to salt intake, were generated from the DR for each meal. The food items analyzed in this study were selected primarily based on dietary behavior recommendations for salt reduction, not solely based on their sodium content, to examine the association between recommended food types and salt intake. Prior to analysis, the food items were selected with reference to recommendations from major public health organizations, such as the WHO and the American Heart Association [13–16, 18, 19, 51–54]. These recommendations typically advise avoiding certain foods (e.g., “*limit the consumption of processed foods*” [13]) or consuming more of others (e.g., “*use more herbs and spices*” [16]). Examples of food-related recommendations are presented in Table S1. We included both foods that should be avoided to reduce salt intake (i.e., soup, pickles, processed foods, and salt-based seasonings) and those that are recommended to support reduced salt intake (i.e., reduced-salt seasonings, herbs and spices, citrus juice and vinegar, fruit, and vegetables). Staple foods (e.g., rice, bread, and noodles) and alcoholic beverages were also included in the analysis, not for their sodium content per se, but because sodium intake at meals has been found to vary depending on the dietary patterns associated with different staple foods and the amount of alcoholic beverages consumed by Japanese adults [23].

Examples of the food items included in each food type variable and how the variables were generated are presented in Table 1. Staple foods were first identified based on the food code assigned to each ingredient using the STFCJ [46] and the name of the dish (e.g., *sushi*). Thereafter, the staple food type for each meal was categorized as rice, bread, noodles, other grain foods, or no staple food [55]. If a meal contained more than one staple food, the staple food with the greatest weight consumed was selected as the staple food for that meal. Soups were identified from the DRs using specific keywords in dish names (e.g., *siru*—meaning “soup” in Japanese) based on previous DR data (5509 days in total) from different Japanese adult populations [56] and popular Japanese recipe websites [57, 58]. Noodle soup was excluded from the soup category and classified as part of the noodle dishes. If a soup dish was consumed during a meal in any amount greater than 0 g, it was categorized as “consumed”; otherwise, it was categorized as “not consumed.”

**Table 1:** Examples of food items included in each food type variable

Variables	Examples^a^	How the variables were generated
**Categorical variables**		
Staple foods (rice, bread, noodles, other staple foods, or no staple food)	**Rice**: white rice, rice balls, curry and rice, fried rice, beef bowl, sushi **Bread**: toast, sandwiches, sweet buns, hamburgers, croissants **Noodles**: fried noodles, ramen, udon (thick Japanese wheat noodles), soba (buckwheat noodles), pasta **Other staple foods**: breakfast cereals, okonomiyaki (savory pancake), pizza, pancake, nikuman (steamed bun with meat filling), sweet potatoes, potatoes, corn	**Step 1**: The food codes that were likely to be included in each staple food category were selected (see Table S2). **Step 2**: The dish names containing these food codes were visually assessed. If the name of the dish clearly referred to a certain staple food (e.g., rice for “sushi,” noodles for “ramen”), the dish was classified under that staple food category. **Step 3**: If the type of staple food in a meal was unclear even after visual assessment, all dish and ingredient names in the meal were considered and the meal was classified into the appropriate staple food category. **Step 4**: If a meal contained more than one staple food, the staple food with the greatest weight consumed was considered the staple food for that meal.
Soup^b^ (consumed or not consumed)	Miso soup, suimono (Japanese clear soup), ozoni soup (Japanese New Year’s soup with broth and vegetables), potage, minestrone soup	**Step 1**: Keywords indicating inclusion (e.g., *siru* - meaning “soup ”in Japanese) and exclusion (e.g., *ramen*) in the names of soup dishes were selected from previous dietary data [56] and common Japanese recipe websites [57, 58]. **Step 2**: Dishes containing the inclusion keywords and not containing the exclusion keywords were listed. **Step 3**: If the dietary records indicated that the dish was consumed in amounts greater than 0 g/meal, the meal was categorized as “soup consumed.”
Pickles (consumed or not consumed)	Kimchi, pickled plums, pickled radish, tsukudani (kelp boiled in soy sauce), pickled ginger	Consumption of more than 0 g per meal was categorized as “consumed,” while amounts of 0 g were categorized as “not consumed.”
Fruit^c^ (consumed or not consumed)	Bananas, apples, mandarin oranges, grapes, persimmons, strawberries, kiwi fruit, dried mangoes	Consumption of more than 0 g per meal was categorized as “consumed,” while amounts of 0 g were categorized as “not consumed.”
Reduced-salt seasonings (consumed or not consumed)	Reduced-salt soy sauce, reduced-salt miso	Consumption of more than 0 g/meal was categorized as “consumed,” except for those used in soups. Amounts of 0 g were categorized as “not consumed.”
Herbs and spices (consumed or not consumed)	Spring onion, pepper, garlic, ginger, mustard, wasabi, hot peppers, curry powder	Consumption of more than 0 g per meal was categorized as “consumed,” while amounts of 0 g were categorized as “not consumed.”
Citrus juice and vinegar (consumed or not consumed)	Grain vinegar, rice vinegar, fruit vinegar, lemon juice, Yuzu juice, lime juice	Consumption of more than 0 g per meal was categorized as “consumed,” while amounts of 0 g were categorized as “not consumed.”
Moderately processed meat and seafood^d^ (consumed or not consumed)	**Meat**: pork ham, bacon, ready-to-eat roast pork, ready-to-eat roast beef, corned beef **Seafood**: canned tuna, salted salmon, dried young sardines, spicy pollack roe, canned crab	Consumption of more than 0 g per meal was categorized as “consumed,” while amounts of 0 g were categorized as “not consumed.”
Highly processed meat and seafood^d^ (consumed or not consumed)	**Meat**: pork sausages, ready-to-eat deep-fried chicken, ready-to-eat pork cutlet, pressed ham, chicken nugget **Seafood**: yaki-chikuwa (baked tubular kamaboko), satsuma-age, crab-flavored fish cake, pounded fish cake, fish sausages	Consumption of more than 0 g per meal was categorized as “consumed,” while amounts of 0 g were categorized as “not consumed.”
Alcoholic beverages (consumed or not consumed)	Beer, shochu (distilled alcoholic beverage), wine, Japanese sake, whisky	Consumption of more than 0 g per meal was categorized as “consumed,” while amounts of 0 g were categorized as “not consumed.”
**Continuous variables**		
Salt-based seasonings (g)	Soy sauce, salt, granulated broth, salad dressing, miso, mayonnaise, ketchup	Summation of the consumed weights of selected foods in this category at each meal, except those used in soups.
Vegetables^e^ (g)	Carrot, cabbage, cucumber, tomatoes, lettuce, daikon radish, laver, mushrooms	Summation of the consumed weights of selected foods in this category at each meal

^a^ All food codes used to generate each variable are available in Table S2

^b^Noodle soup was excluded from the soup category and classified as part of the noodle dishes

^c^ Fruit juice, jam, and pickled plums were excluded from this category

^d^ Moderately and highly processed meat and seafood were identified according to the classification framework developed by researchers at the University of North Carolina at Chapel Hill [59]

^e^ Mushrooms and seaweeds were included in this category

Other food items were identified based on food codes, and the sum of the weight consumed for each meal was calculated. When the consumed weights of reduced-salt seasonings and salt-based seasonings were calculated, those included in soups were excluded to avoid duplication. Regarding processed foods, we focused on meat and seafood to avoid overlap with other food groups (e.g., vegetables) in the analysis. Moreover, processed meat and seafood are generally targeted in several recommendations for reduced salt intake [14, 19, 52]. Food codes for moderately and highly processed meat and seafood were identified according to the classification framework developed by researchers at the University of North Carolina at Chapel Hill [59], which is based on the widely used Nova food classification system [60] and was used in a previous analysis of the dataset utilized in the present study [61]. Mushrooms and seaweeds were included in the vegetable category. The distribution of the consumed weights for each food item was assessed, and the results revealed that salt-based seasonings and vegetables were consumed in more than half of the meals. This indicated frequent consumption; therefore, salt-based seasonings and vegetables were treated as continuous variables. Other food items were not consumed in more than half of the meals; therefore, they were dichotomized into “consumed” (> 0 g/meal) and “not consumed” (0 g/meal). The food codes used to generate each food type variable are listed in Table S2.

### *Participant characteristics*

Body mass index (BMI; kg/m^2^) was calculated by dividing body weight (kg) by height squared (m^2^). For participants who could not be measured (*n =* 7), their self-reported height and weight were used for the analysis. Sex was categorized as male or female. Age at the beginning of the survey was calculated in years based on the participant’s date of birth. Living status was classified as either living alone or with others. Education level was divided into four categories: junior high school or high school, junior college or technical school, university or higher, and other. Employment status was categorized as unemployed, student, part-time, or full-time. Annual household income was classified into three categories: < 5, ≥ 5 to < 8, and ≥ 8 million Japanese yen (as of June 10, 2025, 5 million Japanese yen was equal to 34,507 United States dollars, and 8 million yen was equal to 55,212 United States dollars). Residential areas were grouped into six regions: Hokkaido and Tohoku, Kanto, Hokuriku and Tokai, Kinki, Chugoku and Shikoku, or Kyushu and Okinawa. Municipality types were classified into metropolises (government ordinance-designated cities and special wards of Tokyo), other cities, or towns and villages. Regarding medical history, each of the following diseases was self-reported: hypertension, stroke, dyslipidemia, diabetes mellitus, hyperuricemia, liver disease, kidney disease, heart disease, and cancer. Presence of a history of any of these diseases was categorized as “yes,” whereas absence of a history of any of these diseases was classified as “no.”

### *Energy reporting status*

The accuracy of reported energy intake (EI) for each participant was evaluated based on their average daily EI over eight days, including energy from snacks, using Goldberg’s cutoff for the ratio of EI to basal metabolic rate (BMR) [62]. Participants were classified as plausible reporters, under-reporters, or over-reporters of EI depending on whether their ratio fell within, below, or above the 95% confidence limit for agreement between EI: BMR and their physical activity level (PAL). Owing to the lack of an objective measure of physical activity, a PAL of 1.55, representing a sedentary lifestyle, was applied to all participants. BMR was calculated using an equation tailored for Japanese individuals that accounts for height, weight, age, and sex [63, 64].

We determined the upper and lower cutoff values for the agreement between EI: BMR and PAL with 95% confidence limits, taking into account the coefficient of variation in intakes and other components of energy balance. Specifically, we considered a 23% within-subject variation in EI, an 8.5% precision of the estimated BMR relative to the measured BMR, and a 15% between-subject variation in PAL [62]. Based on these parameters, we defined under-reporters, plausible reporters, and over-reporters as those with an EI: BMR of < 1.07, ≥ 1.07 to < 2.25, and ≥ 2.25, respectively [62].

### *Statistical analysis*

Statistical analyses were performed using SAS version 9.4 (SAS Institute Inc., Cary, NC, US). Given that the sex-separated analyses showed similar associations (data not shown), the results are presented for both sexes combined. The average energy intake, absolute salt intake (g/meal), and salt intake density (g/100 kcal per meal) for the entire study sample were calculated using each participant’s average intake over eight days. Descriptive statistics for participant and meal characteristics are reported as means and SDs or numbers and percentages.

Multilevel linear regression was performed to account for multiple observations (meals) within each participant. We employed random intercepts and an unstructured covariance matrix with multiple meals (level 1) nested within individuals (level 2). To account for salt intake relative to meal size, both absolute salt intake and salt intake density were analyzed as continuous dependent variables. The independent variables included meal contexts and food types (level 1 predictors) and participant characteristics (level 2 predictors). Meal context factors included meal type, day type, eating location, eating companions, and seasons, all of which were entered as categorical variables. The food types included all the variables shown in Table 1. To simplify the interpretation of the regression coefficients, consumption of salt-based seasonings and vegetables was divided by the median of their consumption among consumers. Therefore, the regression coefficients for salt-based seasonings and vegetables indicate the change in absolute salt intake and salt intake density if the consumption of each food item increased by the median amount consumed.

Level 2 predictors included age, BMI, EI, sex, living status, education level, employment status, annual household income, smoking status, residential area, municipality, and self-reported medical history. These variables were entered as categorical variables, except for age, BMI, and EI, which were treated as continuous variables. All categorical independent variables were dummy-coded. Level 1 predictors were group-mean centered and level 2 predictors were grand-mean centered for appropriate interpretation of the model parameters [65, 66]. The sample size for each sex was large enough to obtain unbiased estimates of level 2 standard errors [67].

A step-by-step approach was used to construct multilevel linear models in this study. The independent variables at each level were added to the model step by step using the PROC MIXED procedure [68, 69]. First, an intercept-only model (Model 0) with no independent variables was used to calculate the intraclass correlation (ICC) and design effect [65]. The ICC and design effect were 0.11 and 3.48, respectively, for absolute salt intake, and 0.07 and 2.57, respectively, for salt intake density. These values indicate variability in both measures across individuals and highlight the need for a multilevel analysis [65]. Next, Model 1 was fitted to the level 1 predictors to evaluate the associations of meal context and food types with salt intake, assessed as both absolute and density values. Subsequently, Model 2 was fitted to the level 2 predictors to evaluate the associations between individual-level factors and salt intake. Thereafter, Model 3 was fitted to both level 1 and level 2 predictors. The equations for these models are presented in Text S1. The fixed effects of the independent variables were evaluated using regression coefficients with 95% confidence intervals (CIs). The regression coefficients and variances were estimated using the maximum likelihood method because the regression coefficients were of major interest in this study [68]. Akaike’s information criterion (AIC) was used to assess the goodness of fit of each model [65]. Some contextual variables were significantly correlated with each other (Table S3). However, repeated analyses excluding one of the correlated variables at a time (i.e., meal type, day type, and eating location) yielded similar results (data not shown). In addition, multicollinearity among the independent variables in the final model was assessed using the variance inflation factor, and no variable showed multicollinearity problems (variance inflation factor < 5) [70]. To assess the robustness of the results, we conducted sensitivity analyses by excluding under- and over-reporters of EI. Statistical significance was defined as a two-sided *P*-value < 0.05.

## Results

### Characteristics of the study participants and the meals consumed

The characteristics of the study participants are shown in Table 2. The mean age of the participants was 48.4 years (SD: 17.6), and their mean BMI was 22.9 kg/m^2^ (SD: 3.5). Most of the participants lived with others (86.1%) and had full-time jobs (64.9%). The mean absolute salt intake of the participants was 10.23 g/day (SD: 2.67), and the mean salt intake density was 0.51 g/100 kcal of total energy (SD: 0.10). Majority (88.6%) of the participants were classified as plausible reporters of EI, whereas others were categorized as under-reporters (6.5%) and over-reporters (4.9%). The median consumption of salt-based seasonings and vegetables among consumers was 12.5 g per meal and 80.0 g per meal, respectively.

**Table 2:** Characteristics of the 2757 participants

Variables	Values^a^
Age (years)	48.4 ± 17.6
Body height (cm)	162.7 ± 9.0
Body weight (kg)	61.1 ± 12.1
BMI (kg/m^2^)	22.9 ± 3.5
Sex	
Female	1401 (50.8)
Male	1356 (49.2)
Living status	
Living with others	2375 (86.1)
Living alone	382 (13.9)
Education level	
Junior high school or high school	1094 (39.7)
Junior college or technical school	819 (29.7)
University or higher	828 (30.0)
Other	16 (0.6)
Employment status	
Unemployed	510 (18.5)
Student	121 (4.4)
Part-time job	338 (12.3)
Full-time job	1788 (64.9)
Annual household income^b^ (Japanese yen)	
< 5 million	978 (35.5)
≥ 5 to < 8 million	929 (33.7)
≥ 8 million	850 (30.8)
Smoking status	
Current smoker	451 (16.4)
Former smoker	610 (22.1)
Never smoker	1696 (61.5)
Residential area	
Hokkaido and Tohoku	311 (11.3)
Kanto	953 (34.6)
Hokuriku and Tokai	487 (17.7)
Kinki	367 (13.3)
Chugoku and Shikoku	302 (11.0)
Kyushu and Okinawa	337 (12.2)
Municipality type	
Metropolis^c^	693 (25.1)
Other cities	1786 (64.8)
Towns and villages	278 (10.1)
Self-reported medical history	
Hypertension	425 (15.4)
Stroke	27 (1.0)
Dyslipidemia	160 (5.8)
Diabetes mellitus	97 (3.5)
Hyperuricemia	73 (2.6)
Liver disease	16 (0.6)
Kidney disease	10 (0.4)
Heart disease	69 (2.5)
Cancer	57 (2.1)
Any of the above	674 (24.4)
Salt intake (g/day)	10.23 ± 2.67
Salt intake density (g/100 kcal)	0.51 ± 0.10
Energy intake (kcal/day)	2005 ± 462
Energy reporting status^d^	
Underreporting	180 (6.5)
Plausible reporting	2443 (88.6)
Overreporting	134 (4.9)

^a^ Values are means ± standard deviations or *n* (%)

^b^ On June 10, 2025, 5 million Japanese yen was worth 34,507 US dollars and 8 million yen was worth 55,212 US dollars

^c^ Government ordinance-designated cities and special wards of Tokyo

^d^ Underreporting, plausible reporting, and overreporting were defined as participants with a reported energy intake to basal metabolic rate ratio of < 1.07, ≥ 1.07 to < 2.25, and ≥ 2.25, respectively

Table 3 shows the characteristics of the meals consumed by the participants. Meals were almost evenly distributed according to meal types (breakfast, lunch, and dinner), day types (working or school days and non-working or non-school days), and the four seasons. The most common meal contexts were eating at home (75.5%) and eating alone (41.2%). Rice was the major staple food consumed (58.5%), and approximately one-third of meals were accompanied by soup (30.9%). Use of reduced-salt seasonings was uncommon (1.3%), whereas herbs and spices were commonly used (60.3%).

**Table 3:** Characteristics of 63,239 meals recorded in the eight-day dietary records of the 2757 Japanese adults

Variables	*n*	%^a^
**Meal context**		
Meal type		
Breakfast	19890	31.5
Lunch	21488	34.0
Dinner	21861	34.6
Day type		
Working or school days	33700	53.3
Non-working or non-school days	29539	46.7
Eating location		
At home	47731	75.5
Restaurant	4165	6.6
Other places^b^	11343	17.9
Eating companion		
Alone	26064	41.2
With one person	20076	31.8
With two or more people	17099	27.0
Season		
Spring	15815	25.0
Summer	15797	25.0
Fall	15811	25.0
Winter	15816	25.0
**Food type**		
Staple food		
No staple food	5174	8.2
Rice	37017	58.5
Bread	10947	17.3
Noodles	8627	13.6
Other staple foods	1474	2.3
Other foods		
Soup		
Not consumed	43732	69.2
Consumed	19507	30.9
Pickles		
Not consumed	53569	84.7
Consumed	9670	15.3
Fruit		
Not consumed	48922	77.4
Consumed	14317	22.6
Reduced-salt seasonings^c^		
Not consumed	62435	98.7
Consumed	804	1.3
Herbs and spices		
Not consumed	25117	39.7
Consumed	38122	60.3
Citrus juice and vinegar		
Not consumed	55898	88.4
Consumed	7341	11.6
Moderately processed meat and seafood		
Not consumed	46818	74.0
Consumed	16421	26.0
Highly processed meat and seafood		
Not consumed	51225	81.0
Consumed	12014	19.0
Alcoholic beverages		
Not consumed	55817	88.3
Consumed	7422	11.7

^a^ Some percentages do not total 100 because of rounding

^b^ Examples of other places include workplaces, schools, nursing-care facilities, parks, cars, and other people’s houses

^c^ Except for those used in soups

### Meal context and food types that contribute to salt intake at meals

Table 4 shows the associations between meal- and individual-level factors and absolute salt intake (g) per meal. The ICC value of the null model (Model 0) indicated that 11% of the variance in absolute salt intake at meals was explained by between-individual variability, whereas 89% was explained by within-individual variability across different meals. The fixed effects of each variable observed in Models 1 (meal-level factors) and 2 (individual-level factors) were consistent in direction and magnitude with those observed in Model 3 (meal- and individual-level factors). Model 3 had a lower AIC score than Models 1 and 2, indicating better goodness-of-fit.

**Table 4:** Associations between meal context and food types in relation to salt intake (g/meal)^a^

	Model 0 (Null model)	Model 1 (Meal-level factors)	Model 2 (Individual-level factors)	Model 3 (Meal- and individual-level factors)
**Intercept**	3.48 (3.45, 3.52)^***^	3.49 (3.45, 3.52)^***^	3.49 (3.46, 3.51)^***^	3.49 (3.47, 3.52)^***^
**Meal context**				
Meal type (ref: breakfast)				
Lunch		0.47 (0.44, 0.51)^***^		0.47 (0.44, 0.51)^***^
Dinner		0.84 (0.80, 0.88)^***^		0.84 (0.80, 0.88)^***^
Day type (ref: working or school days)				
Non-working or non-school days		0.10 (0.06, 0.13)^***^		0.10 (0.06, 0.13)^***^
Eating location (ref: at home)				
Restaurant		0.40 (0.34, 0.45)^***^		0.40 (0.34, 0.45)^***^
Other places^b^		-0.17 (-0.21, -0.13)^***^		-0.17 (-0.21, -0.13)^***^
Eating companion (ref: alone)				
One other person		0.08 (0.05, 0.12)^***^		0.08 (0.05, 0.12)^***^
Two or more people		-0.01 (-0.05, 0.02)		-0.01 (-0.05, 0.02)
Season (ref: spring)				
Summer		-0.05 (-0.09, -0.02)^**^		-0.05 (-0.09, -0.02)^**^
Fall		0.09 (0.06, 0.13)^***^		0.09 (0.06, 0.13)^***^
Winter		0.10 (0.07, 0.13)^***^		0.10 (0.07, 0.13)^***^
**Food type**				
Staple food (ref: no staple food)				
Rice		0.25 (0.20, 0.30)^***^		0.25 (0.20, 0.30)^***^
Bread		0.39 (0.33, 0.45)^***^		0.39 (0.33, 0.45)^***^
Noodles		2.29 (2.23, 2.36)^***^		2.29 (2.23, 2.36)^***^
Other staple foods		0.16 (0.06, 0.25)^**^		0.16 (0.06, 0.25)^**^
Other foods				
Soup (consumed; ref: not consumed)		1.06 (1.03, 1.09)^***^		1.06 (1.03, 1.09)^***^
Pickles (consumed; ref: not consumed)		0.72 (0.68, 0.75)^***^		0.72 (0.68, 0.75)^***^
Fruit (consumed; ref: not consumed)		-0.12 (-0.15, -0.09)^***^		-0.12 (-0.15, -0.09)^***^
Reduced-salt seasonings^c^ (consumed; ref: not consumed)		0.35 (0.23, 0.47)^***^		0.35 (0.23, 0.47)^***^
Herbs and spices (consumed; ref: not consumed)		0.13 (0.10, 0.16)^***^		0.13 (0.10, 0.16)^***^
Citrus juice and vinegar (consumed; ref: not consumed)		0.30 (0.26, 0.34)^***^		0.30 (0.26, 0.34)^***^
Moderately processed meat and sea-food (consumed; ref: not consumed)		0.59 (0.56, 0.62)^***^		0.59 (0.56, 0.62)^***^
Highly processed meat and seafood consumed (consumed; ref: not consumed)		0.58 (0.55, 0.61)^***^		0.58 (0.55, 0.61)^***^
Alcoholic beverages (consumed; ref: not consumed)		0.36 (0.32, 0.41)^***^		0.36 (0.32, 0.41)^***^
Salt-based seasonings^c^ (continuous, unit^d^: 12.5 g/meal)		0.35 (0.34, 0.36)^***^		0.35 (0.34, 0.36)^***^
Vegetables^e^ (continuous, unit^d^: 80.0 g/meal)		0.38 (0.36, 0.39)^***^		0.38 (0.36, 0.39)^***^
**Individual-level factors**				
Age (years)			0.003 (0.0005, 0.005)^*^	0.002 (0.0003, 0.005)^*^
Body mass index (kg/m^2^)			0.01 (0.01, 0.02)^***^	0.01 (0.01, 0.02)^***^
Energy intake (kcal/day)			0.001 (0.001, 0.001)^***^	0.001 (0.001, 0.001)^***^
Sex (ref: male)				
Female			-0.14 (-0.21, -0.08)^***^	-0.15 (-0.22, -0.08)^***^
Living status (ref: living with others)				
Living alone			0.06 (-0.02, 0.14)	0.06 (-0.02, 0.14)
Education level (ref: junior high school or high school)				
Junior college or technical school			-0.004 (-0.07, 0.06)	-0.004 (-0.07, 0.06)
University or higher			-0.04 (-0.11, 0.03)	-0.04 (-0.11, 0.03)
Other			-0.08 (-0.42, 0.25)	-0.08 (-0.43, 0.26)
Employment status (ref: unemployed)				
Student			-0.11 (-0.29, 0.06)	-0.12 (-0.29, 0.06)
Part-time job			-0.01 (-0.11, 0.08)	-0.01 (-0.11, 0.08)
Full-time job			-0.04 (-0.12, 0.05)	-0.03 (-0.12, 0.05)
Annual household income^f^ (ref : < 5 million Japanese yen)				
≥ 5 to < 8 million			-0.07 (-0.13, 0.002)^*^	-0.07 (-0.13, 0.002)^*^
≥ 8 million			-0.02 (-0.09, 0.05)	-0.02 (-0.09, 0.06)
Smoking status (ref: current smoker)				
Former smoker			-0.22 (-0.31, -0.14)^***^	-0.23 (-0.32, -0.15)^***^
Never smoker			-0.22 (-0.29, -0.14)^***^	-0.23 (-0.30, -0.15)^***^
Residential area (ref: Hokkaido and Tohoku)				
Kanto			-0.03 (-0.11, 0.06)	-0.02 (-0.11, 0.07)
Hokuriku and Tokai			-0.13 (-0.23, -0.03)^**^	-0.13 (-0.23, -0.03)^**^
Kinki			-0.23 (-0.34, -0.13)^***^	-0.23 (-0.33, -0.12)^***^
Chugoku and Shikoku			-0.15 (-0.26, -0.04)^**^	-0.14 (-0.25, -0.03)^*^
Kyushu and Okinawa			-0.30 (-0.41, -0.20)^***^	-0.30 (-0.41, -0.19)^***^
Municipality type (ref: metropolis^g^)				
Other cities			-0.01 (-0.07, 0.05)	-0.01 (-0.07, 0.06)
Towns and villages			0.01 (-0.09, 0.10)	0.01 (-0.09, 0.10)
Self-reported medical history^h^ (ref: no)				
Yes			0.02 (-0.05, 0.09)	0.02 (-0.05, 0.09)
**Variance components (random effects)**				
Level 2 intercept	0.60^***^	0.71^***^	0.26^***^	0.38^***^
Residual	4.68^***^	2.24^***^	4.69^***^	2.25^***^
**Model Summary**				
Akaike information criterion	280895	236459	279468	235057
Intraclass correlation coefficient	0.11	0.24	0.05	0.14
Design effect	3.48	6.29	2.14	4.15

Ref, reference category

^a^ The dependent variable was absolute salt intake (g) per meal. Regression coefficients with 95% confidence intervals (in parentheses) are shown for meal context, food types, and individual characteristics. Other values are parameter estimates, with 95% confidence intervals indicated in parentheses if available. The regression coefficients represent the change in absolute salt intake at meals for a one-unit increase in consumption of salt-based seasonings and vegetables, age, body mass index, and energy intake. For other independent variables, the regression coefficients represent the difference in absolute salt intake at meals compared to the reference category. ^*^P < 0.05, ^**^P < 0.01, ^***^P < 0.001

^b^ Examples of other places include workplaces, schools, nursing-care facilities, parks, cars, and other people’s houses

^c^ Except for those used in soups

^d^ The values were determined based on the median intake of each food item among consumers across all meals

^e^ Mushrooms and seaweeds were also included in this category

^f^ On June 10, 2025, 5 million Japanese yen was worth 34,507 US dollars, and 8 million yen was worth 55,212 US dollars

^g^ Government ordinance-designated cities and special wards of Tokyo

^h^ At least one of the following: hypertension, stroke, dyslipidemia, diabetes mellitus, hyperuricemia, liver disease, kidney disease, heart disease, and cancer

The final model (Model 3) revealed significant associations between absolute salt intake (g/meal) and all meal context factors. Absolute salt intakes at lunch (*β*: 0.47, 95% CI: 0.44, 0.51) and dinner (*β*: 0.84, 95% CI: 0.80, 0.88) were higher than at breakfast. In addition, absolute salt intake on non-working or non-school days was higher (*β*: 0.10, 95% CI: 0.06, 0.13) than that on working or school days. Furthermore, eating at restaurants was associated with higher absolute salt intake (*β*: 0.40, 95% CI: 0.34, 0.45) than eating at home, whereas eating at other places, such as workplaces and schools, was associated with lower absolute salt intake than eating at home (*β*: -0.17, 95% CI: -0.21, -0.13). Additionally, eating with one other person was associated with higher absolute salt intake (*β*: 0.08, 95% CI: 0.05, 0.12) than eating alone. In addition, absolute salt intake from meals consumed in summer was lower than that from meals consumed in spring (*β*: -0.05, 95% CI: -0.09, -0.02). However, absolute salt intakes in fall (*β*: 0.09, 95% CI: 0.06, 0.13) and winter (*β*: 0.10, 95% CI: 0.07, 0.13) were higher than that in spring.

All food types were associated with absolute salt intake per meal. Eating staple foods was associated with higher absolute salt intake than not eating them, with noodles having the highest coefficient (*β*: 2.29, 95% CI: 2.23, 2.36). Moreover, meals containing soup, pickles, moderately and highly processed meat and seafood, or alcoholic beverages were associated with higher absolute salt intake than meals without these foods. Consuming reduced-salt seasonings (*β*: 0.35, 95% CI: 0.23, 0.47), herbs and spices (*β*: 0.13, 95% CI: 0.10, 0.16), and citrus juice and vinegar (*β*: 0.30, 95% CI: 0.26, 0.34) were also associated with higher absolute salt intake than not consuming them. In addition, consumption of salt-based seasonings (*β*: 0.35, 95% CI: 0.34, 0.36) and vegetables (*β*: 0.38, 95% CI: 0.36, 0.39) was positively associated with absolute salt intake. In contrast, consuming fruit was associated with lower absolute salt intake (*β*: -0.12, 95% CI: -0.15, -0.09).

Except for a few variables, similar results were obtained when the dependent variable was salt intake density (g/100 kcal per meal) (Table 5). Contrary to the results for absolute salt intake (g), eating at restaurants was associated with lower salt intake density (*β*: -0.01, 95% CI: -0.02, -0.0004) than eating at home. Additionally, no associations were observed between salt intake density and either eating with one other person or eating in summer, while eating with two or more people was associated with low sodium intake density. Furthermore, eating rice, bread, or other staple foods, as well as meals containing herbs and spices or alcoholic beverages, was associated with lower salt intake density. Sensitivity analyses, excluding under- and over-reporters of EI, provided similar results for both absolute salt intake (Table S4) and salt intake density (Table S5).

**Table 5:** Meal context and food types in relation to salt intake density (g/100 kcal) of 2757 Japanese adults^a^

	Model 0 (Null model)	Model 1 (Meal-level factors)	Model 2 (Individual-level factors)	Model 3 (Meal- and individual-level factors)
**Intercept**	0.56 (0.56, 0.57)^***^	0.56 (0.56, 0.57)^***^	0.56 (0.56, 0.57)^***^	0.56 (0.56, 0.57)^***^
**Meal context**				
Meal type (ref: breakfast)				
Lunch		0.03 (0.02, 0.03)^***^		0.03 (0.02, 0.03)^***^
Dinner		0.01 (0.004, 0.02)^**^		0.01 (0.004, 0.02)^**^
Day type (ref: working or school days)				
Non-working or non-school days		0.01 (0.0003, 0.01)^*^		0.007 (0.0003, 0.01)^*^
Eating location (ref: at home)				
Restaurant		-0.01 (-0.02, -0.00004)^*^		-0.01 (-0.02, -0.00004)^*^
Other places^b^		-0.01 (-0.02, -0.005)^**^		-0.01 (-0.02, -0.005)^**^
Eating companion (ref: alone)				
One other person		0.002 (-0.005, 0.01)		0.002 (-0.005, 0.009)
Two or more people		-0.01 (-0.02, -0.004)^**^		-0.01 (-0.02, -0.004)^**^
Season (ref: spring)				
Summer		-0.002 (-0.01, 0.004)		-0.002 (-0.008, 0.004)
Fall		0.01 (0.004, 0.02)^**^		0.01 (0.004, 0.02)^**^
Winter		0.01 (0.003, 0.02)^**^		0.009 (0.003, 0.02)^**^
**Food type**				
Staple food (ref: no staple food)				
Rice		-0.11 (-0.12, -0.10)^***^		-0.11 (-0.12, -0.10)^***^
Bread		-0.11 (-0.12, -0.10)^***^		-0.11 (-0.12, -0.10)^***^
Noodles		0.34 (0.33, 0.35)^***^		0.34 (0.33, 0.35)^***^
Other staple foods		-0.15 (-0.17, -0.14)^***^		-0.15 (-0.17, -0.14)^***^
Other foods				
Soup (consumed; ref: not consumed)		0.21 (0.20, 0.21)^***^		0.21 (0.20, 0.21)^***^
Pickles (consumed; ref: not consumed)		0.10 (0.09, 0.10)^***^		0.10 (0.09, 0.10)^***^
Fruit (consumed; ref: not consumed)		-0.09 (-0.10, -0.09)^***^		-0.09 (-0.10, -0.09)^***^
Reduced-salt seasonings^c^ (consumed; ref: not consumed)		0.03 (0.01, 0.05)^**^		0.03 (0.01, 0.05)^**^
Herbs and spices (consumed; ref: not consumed)		-0.01 (-0.01, -0.003)^**^		-0.01 (-0.01, -0.003)^**^
Citrus juice and vinegar (consumed; ref: not consumed)		0.03 (0.03, 0.04)^***^		0.03 (0.03, 0.04)^***^
Moderately processed meat and sea-food (consumed; ref: not consumed)		0.06 (0.06, 0.07)^***^		0.06 (0.06, 0.07)^***^
Highly processed meat and seafood consumed (consumed; ref: not consumed)		0.03 (0.03, 0.04)^***^		0.03 (0.03, 0.04)^***^
Alcoholic beverages (consumed; ref: not consumed)		-0.08 (-0.09, -0.07)^***^		-0.08 (-0.09, -0.07)^***^
Salt-based seasonings^c^ (continuous, unit^d^: 12.5 g/meal)		0.03 (0.03, 0.03)^***^		0.03 (0.03, 0.03)^***^
Vegetables^e^ (continuous, unit^d^: 80.0 g/meal)		0.02 (0.01, 0.02)^***^		0.02 (0.01, 0.02)^***^
**Individual-level factors**				
Age (years)			0.001 (0.0004, 0.001)^***^	0.0008 (0.0004, 0.001)^***^
Body mass index (kg/m^2^)			0.001 (-0.0002, 0.002)	0.001 (-0.0002, 0.002)
Energy intake (kcal/day)			-0.00004 (-0.00005, -0.00003)***	-0.00004 (-0.00005, -0.00003)***
Sex (ref: male)				
Female			0.01 (0.003, 0.02)*	0.01 (0.003, 0.02)*
Living status (ref: living with others)				
Living alone			-0.01 (-0.02, 0.01)	-0.01 (-0.02, 0.01)
Education level (ref: junior high school or high school)				
Junior college or technical school			0.0008 (-0.01, 0.01)	0.001 (-0.01, 0.01)
University or higher			-0.004 (-0.02, 0.01)	-0.004 (-0.02, 0.01)
Other			0.0002 (-0.06, 0.06)	0.0006 (-0.05, 0.06)
Employment status (ref: unemployed)				
Student			-0.01 (-0.04, 0.02)	-0.01 (-0.04, 0.02)
Part-time job			-0.02 (-0.03, 0.007)*	-0.02 (-0.03, -0.002)*
Full-time job			-0.03 (-0.04, -0.01)***	-0.03 (-0.04, -0.01)***
Annual household incomef (ref : < 5 million Japanese yen)				
≥ 5 to < 8 million			-0.005 (-0.02, 0.01)	-0.005 (-0.015, 0.006)
≥ 8 million			0.0003 (-0.01, 0.01)	0.0002 (-0.011, 0.012)
Smoking status (ref: current smoker)				
Former smoker			-0.01 (-0.03, -0.0007)*	-0.01 (-0.03, -0.001)*
Never smoker			-0.01 (-0.02, 0.0004)	-0.01 (-0.02, 0.001)
Residential area (ref: Hokkaido and Tohoku)				
Kanto			-0.01 (-0.03, -0.0003)*	-0.01 (-0.03, -0.000003)*
Hokuriku and Tokai			-0.02 (-0.04, -0.01)**	-0.02 (-0.04, -0.01)**
Kinki			-0.06 (-0.07, -0.04)***	-0.06 (-0.07, -0.04)***
Chugoku and Shikoku			-0.04 (-0.05, -0.02)***	-0.04 (-0.05, -0.02)***
Kyushu and Okinawa			-0.05 (-0.07, -0.03)***	-0.05 (-0.07, -0.03)***
Municipality type (ref: metropolisg)				
Other cities			0.01 (-0.004, 0.02)	0.01 (-0.004, 0.02)
Towns and villages			0.02 (0.0002, 0.03)*	0.02 (0.0002, 0.03)*
Self-reported medical historyh (ref: no)				
Yes			0.01 (-0.004, 0.02)	0.01 (-0.004, 0.02)
**Variance components (random effects)**				
Level 2 intercept	0.01***	0.01***	0.01***	0.01***
Residual	0.11***	0.08***	0.11***	0.08***
**Model Summary**				
Akaike information criterion	45044	19836	44824	19618
Intraclass correlation coefficient	0.07	0.12	0.06	0.11
Design effect	2.57	3.69	2.35	3.40

Ref, reference category

^a^ A total of 63,239 meals were analyzed. The dependent variable was salt intake density (g/100 kcal) per meal. Regression coefficients with 95% confidence intervals (in parentheses) are shown for meal context and food types and individual characteristics. Other values are parameter estimates, with 95% confidence intervals indicated in parentheses if available. The regression coefficients represent the change in salt intake density at meals for a one-unit increase in salt-based seasonings and vegetables, age, body mass index, and energy intake. For other independent variables, the regression coefficients represent the difference in salt intake density at meals compared to the reference category. ^*^P < 0.05, ^**^P < 0.01, ^***^P < 0.001

^b^ Examples of other places include workplaces, schools, nursing-care facilities, parks, cars, and other people’s houses

^c^ Except for those used in soups

^d^ The values were determined based on the median intake of each food item among consumers across all meals

^e^ Mushrooms and seaweeds were also included in this category

^f^ On June 10, 2025, 5 million Japanese yen was worth 34,507 US dollars, and 8 million yen was worth 55,212 US dollars

^g^ Government ordinance-designated cities and special wards of Tokyo

^h^ At least one of the following: hypertension, stroke, dyslipidemia, diabetes mellitus, hyperuricemia, liver disease, kidney disease, heart disease, and cancer

## Discussion

### Main findings

To the best of our knowledge, this is the first study in which the associations between salt intake at meals and various meal contexts and food types were comprehensively analyzed using EMA. In this large sample of Japanese adults living across various regions, all selected context variables and food types were associated with within-individual variations in salt intake at meals. Specifically, absolute salt intake and salt intake density were both higher at lunch and dinner, on non-working or non-school days, and during fall and winter than at breakfast, on working or school days, and during spring, respectively. Regarding food types, salt intake (both in grams per meal and per 100 kcal per meal) was higher in meals that included noodles, soup, pickles, reduced-salt seasonings, citrus juice and vinegar, or moderately and highly processed meat and seafood, whereas it was lower in meals containing fruit. Additionally, the consumption of salt-based seasonings and vegetables was positively associated with absolute salt intake and salt intake density. These findings remained generally consistent when the analysis was restricted to plausible EI reporters.

### Meal context and salt intake

The associations between meal context, food type, and sodium intake have not been examined in a previous study using EMA. The results of the present study indicate that salt intake (both absolute and density values) at lunch and dinner is higher than that at breakfast. Similar findings have been reported in several previous studies [22–24]. The difference in salt intake according to meal type may be due to variations in food consumption at breakfast, lunch, and dinner [23, 24, 71]. For example, it has been reported that Japanese individuals have higher EI at lunch and dinner than that at breakfast [24, 71]. Additionally, dietary patterns in Japan often include consumption of noodle dishes (e.g., ramen and udon), which have a high salt content and are typically eaten for lunch [23]. We also observed that salt intake at meals on non-working or non-school days was higher than that on working or school days. Similarly, previous studies have reported higher sodium intake on weekends than on weekdays [26, 27]. These findings suggest that salt intake varies depending on daily schedules.

The results of a previous systematic review suggested that people who frequently consume fast foods or restaurant meals have a higher sodium intake than those who do not [25]. Similarly, the results of the present study showed that eating at restaurants was associated with higher absolute salt intake than eating at home. However, eating at restaurants was also associated with lower salt intake density. These findings suggest that although the salt concentration in restaurant meals may not necessarily be higher than that in meals eaten at home, people tend to eat more at restaurants, leading to a higher absolute salt intake. Thus, dietary recommendations regarding avoiding eating out [16, 19] and strategies for reducing salt intake at restaurants [15, 52] seem reasonable.

In the present study, we observed that eating in other places, such as workplaces and schools, was associated with lower absolute salt intake and salt intake density than eating at home. A previous study conducted in China showed no significant difference in sodium intake between people who habitually eat at company or school canteens and those who habitually eat at home [72]. Notably, a questionnaire with an single item on eating location was used in this previous study [72]. In the present study, we could not identify where the participants’ meals were prepared due to a lack of sufficient information; however, our results suggest that meals consumed at home may not always be healthy in terms of salt content. Additional studies conducted using EMA will help clarify whether there is a direct association between eating location and salt intake.

Previous studies have shown that eating with others is linked to sodium intake [28, 73]. For instance, meals eaten with someone else had a lower mean Healthy Eating Index score for sodium than those eaten alone [28]. Similarly, the results of the present study indicated that eating with one other person was associated with a slightly higher absolute salt intake. However, no association was observed for salt intake density, suggesting that the higher intake may be attributed to larger meal sizes rather than greater salt content per unit of energy. It is well known that eating with others can affect food choices and portion sizes owing to social factors such as modeling eating behavior and impression management [74–76]. However, the reason why eating with two or more people was not associated with salt intake in the present study remains unclear. Further research is needed to examine whether and how the number of eating companions affects salt intake.

We observed that meals in summer were associated with lower absolute salt intake, while meals in fall and winter were associated with both higher absolute salt intake and higher salt intake density compared with spring. This seasonal variation may be due to differences in food consumption across seasons [77, 78]. Notably, seasonal variations in sodium intake have been observed in Japan [79], Switzerland [80], and China [78] but not in the United States [81]. This inconsistency in findings may be due to variations in the effects of seasons on eating behavior and food availability from country to country [82].

### Food type and salt intake

In this study, eating staple foods was associated with higher absolute salt intake, regardless of the type of staple food. This may reflect not only the salt content of the staple foods themselves but also the foods eaten with them [23]. However, rice, bread, and other staple foods were associated with lower salt intake density, suggesting that meals containing these staples may be relatively low in sodium density. Given that a typical Japanese meal consists of a staple food (e.g., rice, bread, and noodles), main dishes, and side dishes [23, 55, 71], avoiding staple foods to reduce salt intake is neither practical nor recommended. Instead, the type of staple food and main and side dishes should be carefully considered. Specifically, our results showed that consuming noodles was associated with both higher absolute salt intake and higher salt intake density. In Japan, “*leave noodle soup*” is a common recommendation for reduction of salt intake [19]. However, considering that sodium is present not only in noodle soup but in many types of noodles themselves as well [46], reducing the frequency or amount of noodle consumption may be more effective in lowering sodium intake.

A previous study indicated that higher alcohol consumption is associated with greater salt intake in Japanese adults [29]. Consistent with this, the results of the present study showed that the participants had higher absolute salt intake when they consumed alcoholic beverages than when they did not. However, meals that included alcoholic beverages were associated with lower salt intake density, suggesting that individuals may consume larger quantities of food when drinking, resulting in a higher absolute salt intake. Although the salt-reduction strategies examined in this study [13–16, 18, 19, 51–54] do not include any recommendations regarding alcohol consumption, limiting the consumption of alcoholic beverages may be effective in reducing sodium intake, considering their association with salt preference [83].

Avoiding salty foods, such as soups, pickles, processed foods, and salt-based seasonings, is a major dietary recommendation for reducing salt intake [13–16, 18, 19, 51–54]. In the present study, meals containing these foods were associated with higher salt intake, both in absolute terms and per energy unit. Although several salt-reduction strategies focus on limiting the consumption of highly or ultra-processed foods [53, 54], our results showed that moderately processed meat and seafood also contributed to salt intake at meals. This finding is understandable, as moderately processed foods include various salty products, such as dried fish, which is recommended to be consumed in moderation in Japan [19]. Thus, reducing the consumption of both highly and moderately processed meat and seafood may help reduce salt intake.

Several behavioral strategies for reducing salt intake from seasonings recommend using reduced-salt seasonings, herbs and spices, and citrus juice and vinegar [13, 14, 16, 18, 19, 51–53]. Previous studies have shown that food acids, such as citric and acetic acids, can enhance the perception of saltiness [84]. Similarly, herbs and spices have been reported to enhance flavor and help lower sodium intake in ways that are acceptable to consumers [85]. However, the results of this study showed that meals containing reduced-salt seasonings, herbs and spices, or citrus juice and vinegar are associated with higher absolute salt intak*e.* As this was a cross-sectional study, whether these foods actually increased salt intake at meals or the participants used them because they felt that the meal was salty remains unclear. In addition, given that meals containing herbs and spices were associated with a lower salt intake density, their use may have led to increased overall food consumption, resulting in higher overall salt intake. Thus, it may be premature to conclude that these ingredients are ineffective in reducing salt intake based on the present findings.

Previous studies have shown that higher vegetable consumption is associated with higher sodium intake in Japan [30, 31]. Similarly, the results of this study indicated that an increase of 80 g in vegetable consumption was associated with a higher salt intake of 0.38 g/meal and 0.02 g/100 kcal per meal, independent of the amount of salt-based seasoning consumed or whether other salty foods (i.e., soup and pickles) were consumed. The exact reason for this finding is unknown; however, salt from the vegetable products themselves (e.g., canned vegetables) or foods not included in the analyses but eaten with vegetables (e.g., cheese) may have contributed to salt intake. Moreover, we did not consider the amount consumed or salt concentration in the foods analyzed, except for salt-based seasonings. Therefore, it is plausible that the consumption of vegetables increased the amount of salty foods consumed or led to the selection of foods with higher salt content, resulting in higher salt intake. Given that vegetable consumption among most Japanese people has not reached the recommended levels [31], encouraging an increase in vegetable intake is necessary. However, when promoting vegetable consumption, it is advisable to encourage people to pay attention to their salt intake while consuming vegetables. As with vegetables, consuming fruits is recommended for reduction of salt intake [14, 16]. In support of this recommendation, eating fruit was associated with a lower salt intake, –0.12 g/meal and –0.09 g /100 kcal per meal, in this study. As fruits contain little salt, replacing dishes seasoned with salt with fruit may reduce the overall salt content of meals.

### Strengths and limitations

The strength of this study is the use of detailed information based on the eight-day DRs of a large sample of Japanese adults residing in geographically diverse locations. Additionally, the DRs were completed on weekdays and weekends across all four seasons, allowing for the analysis and depiction of changes in dietary behaviors and meal environments depending on daily schedules and seasonal variations. Moreover, the use of EMA enabled us to observe within-person dynamic relationships between meal contexts, food types, and salt intake across different meals in the natural settings of daily life.

However, this study has several limitations that should be acknowledged. First, although the goal of the participant recruitment was to reflect regional population distributions, we used snowball sampling, a type of non-probability sampling technique, for the recruitment. This method may have introduced selection bias and limited the generalizability of the findings, as the study sample comprised volunteers who may have been more health-conscious than the general population. The participants in this study had a relatively high level of education and annual household income, whereas their height, weight, and BMI were similar to those of a nationally representative sample [41, 86]. Nevertheless, future research should ideally be conducted in a more representative population to enhance generalizability. Second, dietary intake was self-reported through the DRs, which may have been affected by misreporting due to reactivity and social desirability bias [87]. In addition, event-based diaries may involve some degree of retrospection if entries are made after the eating event [33]. Although participants were instructed to record their dietary intake at each meal in accordance with the EMA approach, we cannot deny the possibility that some completed the records at the end of the day, which could induce recall bias [33]. Additionally, when food weight data were missing, research dietitians estimated the values. Participants were required to report the names of seasonings used but were not asked to provide their measured weights. During the development of the original survey dataset, the number of corrections or additions made by the dietitians was not recorded. These limitations may have contributed to the misestimation of salt intake and its association with meal contexts and food types. Although 24-hour urine collection is the gold standard for the assessment of sodium intake [88], it was not feasible for the sample size of this study. Moreover, there are no biomarkers of dietary intake at the meal level. In Japan, a previous study conducted a thorough evaluation of salt intake using a four-day weighed DR, with the main purpose of estimating salt intake, including detailed measurement of seasonings [2]. Despite differences in data collection methods, the estimates of salt intake in present study were very similar to those reported in that previous study, with absolute intake at 10.2 g/day (SD: 2.7) vs. 10.2 g/day (SD: 2.9) [2], and salt intake density at 0.5 g/100 kcal (SD: 0.1) vs. 0.5 g/100 kcal (SD: 0.1) [2]. Additionally, most of the participants were classified as plausible EI reporters, and their results were consistent with those of the total sample population. Given that the reporting accuracy of EI is correlated with that of sodium intake [89], misreporting of sodium intake was not considered to have greatly affected the findings of this study. Finally, we did not account for other time-varying factors that may affect salt intake, such as mood, physiological state, or emotions [36, 90]. More time-intensive research is needed to understand how these factors affect dietary behavior and salt intake throughout the day.

### Future implications

This study revealed that all the meal context factors examined were associated with salt intake. Specifically, special attention may be needed to reduce salt intake from meals consumed at lunch and dinner. In addition, limiting the consumption of noodles, soup, pickles, moderately and highly processed meat and seafood, alcoholic beverages, and salt-based seasonings, while increasing the consumption of fruit, may be particularly effective in reducing salt intake. Additionally, this study showed that certain food types recommended for sodium reduction, such as consuming reduced-salt seasonings, herbs and spices, citrus juice and vinegar, and vegetables, may paradoxically be associated with higher absolute salt intake. Food-related decisions are made repeatedly within a day, and their accumulated results can contribute considerably to the overall success of reduction in salt intake [36]. Our findings highlight the relative contribution of specific food types, often included in public health strategies for sodium reduction, to salt intake, and will be helpful for developing realistic, meaningful, and practical behavioral strategies that can effectively reduce salt intake [91].

Owing to the cross-sectional design of this study, causal relationships between salt intake and contextual factors or food types could not be established. Additionally, we did not examine the participants’ knowledge and attitudes toward reduced salt intake or their motivations for making food choices. Therefore, it remains unclear whether consuming the food types analyzed in this study with the intention of reducing salt intake is effective. Further interventional studies and in-depth research on the reasons underlying food consumption are needed to investigate how the food types analyzed in this study may contribute to reducing salt intake. Moreover, as the findings of this study were obtained in a Japanese population with distinct dietary patterns [92, 93], and snacks were excluded from the analysis, generalizability may be limited in cultures where food types, eating habits, or the contribution of snacks differ substantially. Therefore, similar analyses in different countries or regions are warranted.

## Conclusions

This study demonstrated that various meal contexts and food types, including what and how much was consumed and where, when, and with whom the meal was consumed, were associated with within-individual variations in salt intake from meals in a Japanese adult population. Our findings, which were obtained using EMA techniques, can help advance the understanding of the microcontextual and intra-individual factors related to salt intake and identify effective behavioral strategies for reduction of salt intake. Further research is warranted for a better understanding of the relevant modifiable factors associated with salt intake in order to facilitate the development of effective strategies for reduction of salt intake.

## Electronic supplementary material

Below is the link to the electronic supplementary material.

Supplementary Material 1

## Data Availability

The datasets generated and analysed during the current study are not publicly available due to restrictions imposed by the Ministry of Health, Labour, and Welfare, Japan.

## Abbreviations

AIC: Akaike’s information criterion
BMI: Body mass index
BMR: Basal metabolic rate
CI: Confidence interval
DR: Dietary record
EI: Energy intake
EMA: Ecological momentary assessment
ICC: Intraclass correlation
MINNADE: MINistry of health, labour and welfare-sponsored NAtionwide study on Dietary intake Evaluation
PAL: Physical activity level
SD: Standard deviation
STFCJ: Standard Tables of Food Composition in Japan
STROBE: Strengthening the Reporting of Observational Studies in Epidemiology
WHO: World Health Organization
