# Supplementary material for: Ecological momentary assessment of meal context and food types contributing to salt intake at meals
Source: Int J Behav Nutr Phys Act. 2025 Jun 28;22:85. doi: 10.1186/s12966-025-01780-1 (PMC12205499; doi:10.1186/s12966-025-01780-1)
Supplement: Supplementary file 1 — Supplementary Material 1 [file 12966_2025_1780_MOESM1_ESM.pdf]

Text S1 Model equations for the mixed effects regression models

Model 0:

$Y_{ij} = \gamma_{00} + u_{0j} + r_{ij}$

Y_{ij}: salt intake (g/meal or g/100 kcal) based on meal *i* consumed by participant *j*

γ_{00}: grand-mean salt intake (g/meal or g/100 kcal per meal) of all participants

u_{0j}: participant-specific deviation in salt intake (g/meal or 100 kcal per meal) from the grand mean

r_{ij}: meal-specific deviation in salt intake (g/meal or g/100 kcal) from the mean of participant *j*

Model 1:

$Y_{ij} = \gamma_{00} + \beta_1$Meal type + β_2Day type + β_3Eating location + β_4Eating companion + β_5Season + β_6Staple food + β_7Soup + β_8Pickles + β_9Fruit + β_{10}Reduced-salt seasonings + β_{11}Herbs and spices + β_{12}Citrus juice and vinegar + β_{13}Moderately processed meat and seafood + β_{14}Highly processed meat and seafood+ β_{15}Alcoholic beverages + β_{16}Salt-based seasonings + β_{17}Vegetables + β_{18}Non-alcoholic beverages + u_{0j}+ r_{ij}

Y_{ij}: salt intake (g/meal or g/100 kcal) based on meal *i* consumed by participant *j*

γ_{00}: grand-mean salt intake (g/meal or g/100 kcal per meal) of all participants

β_1–β_{18}: the average effect of each independent variable on salt intake (g/meal or g/100 kcal) at each meal across all participants

u_{0j}: participant-specific deviation in salt intake (g/meal or g/100 kcal per meal) from the grand mean

r_{ij}: meal-specific deviation in salt intake (g/meal or g/100 kcal) from the mean of participant *j*

Categorical independent variables (meal type, day type, eating location, eating companion, season, staple food, soup, pickles, fruits, reduced-salt seasonings, herbs and spices, citrus juice and vinegar, moderately processed meat and seafood, highly processed meat and seafood, and alcoholic beverages) were dummy coded. All independent variables were group-mean centered.

Model 2:

Y_{ij} = γ_{00} + β_{19}Age + β_{20}Body mass index + β_{21}Energy intake + β_{22}Sex + β_{23}Living status + β_{24}Education level + β_{25}Employment status + β_{26}Annual household income + β_{27}Smoking status + β_{28}Region + β_{29}Municipality type + β_{30}Self-reported medical history + u_{0j}+ r_{ij}

Y_{ij}: salt intake (g/meal or g/100 kcal) based on meal *i* consumed by participant *j*

γ_{00}: grand-mean salt intake (g/meal or g/100 kcal per meal) of all participants

β_{19}–β_{30}: the average effect of each independent variable on salt intake (g) at each meal across all participants

u_{0j}: participant-specific deviation in salt intake (g/meal or g/100 kcal per meal) from the grand mean

r_{ij}: meal-specific deviation in salt intake (g/meal or g/100 kcal) from the mean of participant *j*

Categorical independent variables (sex, living status, education level, employment status, annual household income, smoking status, region, municipality type, and self-reported medical history) were dummy coded. All independent variables were grand-mean centered.

Model 3:

Y_{ij} = γ_{00} + β_{1}Meal type + β_{2}Day type + β_{3}Eating location + β_{4}Eating companion + β_{5}Season + β_{6}Staple food + β_{7}Soup + β_{8}Pickles + β_{9}Fruit + β_{10}Reduced-salt seasonings + β_{11}Herbs and spices + β_{12}Citrus juice and vinegar + β_{13}Moderately processed meat and seafood + β_{14}Highly processed meat and seafood+ β_{15}Alcoholic beverages + β_{16}Salt-based seasonings + β_{17}Vegetables + β_{18}Non-alcoholic beverages + β_{19}Age + β_{20}Body mass index + β_{21}Energy intake + β_{22}Sex + β_{23}Living status + β_{24}Education level + β_{25}Employment status + β_{26}Annual household income + β_{27}Smoking status + β_{28}Region + β_{29}Municipality type + β_{30}Self-reported medical history + u_{0j}+ r_{ij}

Y_{ij}: salt intake (g/meal or g/100 kcal) based on meal *i* consumed by participant *j*

γ_{00}: grand-mean salt intake (g/meal or g/100 kcal per meal) of all participants

β_{1}–β_{30}: the average effect of each independent variable on salt intake (g/meal or g/100 kcal) at each meal across all participants

u_{0j}: participant-specific deviation in salt intake (g/meal or g/100 kcal per meal) from the grand mean.

r_{ij}: meal-specific deviation in salt intake (g/meal or g/100 kcal) from the mean of participant *j*

Categorical independent variables were dummy coded. All level 1 independent predictors (variables entered into Model 1) were group-mean centered, whereas level 2 independent predictors (variables entered into Model 2) were grand-mean centered.

Table S1 Food items included in dietary recommendations for reduction of salt intake

Food items	Examples of dietary behavior recommendations for salt reduction[a]
**Foods to be avoided**	
Soup	If you drink miso soup more than twice a day, reduce the amount per serving [19].
Pickles	Avoid pickles [19].<br>Go easy on those "fixins" and salty side dishes such as pickles, pickled vegetables, olives, and sauerkraut [16].
Processed foods	Avoid processed foods containing high levels of salt. Pay attention to dried fish [19].<br>Limit the consumption of processed foods [13].<br>When possible, purchase fresh poultry, fish, pork, and lean meat, rather than cured, salted, smoked, and other processed meats [14].<br>Choose fresh meat, poultry, and seafood, rather than processed varieties [52].<br>Eat mostly fresh food instead of processed food that has had salt added [51].<br>Skip or limit frozen dinners and other high-sodium fare such as pizza, fast food, packaged mixes, and canned soups or broths [16].<br>Prefer fresh and minimally processed foods and avoid ultra-processed foods [53].<br>You can eat less sodium by limiting highly processed foods [54].
Salt-based seasonings	Do not use seasonings unnecessarily. Use seasonings after checking the seasoning [19].<br>Cook with little or no added sodium/salt [13].<br>Limit the amount of table salt you add to foods when cooking, baking, or at the table [52].<br>Cook pasta, rice and hot cereal without salt [15].<br>Limit your use of condiments such as salad dressings, ketchup, barbecue sauce, and hot sauce [16].<br>Limit the amount of salt you add when cooking and eating [18].<br>Try not to use too many sauces that can be high in salt [18].<br>Use small amounts of salt in cooking [53].<br>Use only very small amounts of salty sauces [51].<br>Avoid adding salt at the table [51].
**Foods to be recommended**	
Reduced-salt seasonings	Use low-sodium seasonings [19].<br>Choose low-sodium products [13].<br>Choose light or reduced sodium condiments [52].<br>Combine lower-sodium versions of food with regular versions [15].<br>Go "low or no" with sodium-free or low-sodium foods [16].<br>Try low or reduced-salt versions of foods and sauces [18].
Herbs and spices	Use herbs and spices to flavor food, rather than salt [13].<br>Try no-salt seasoning blends and herbs and spices instead of salt to add flavor to your food [52].<br>Use more herbs and spices [16].<br>Use natural spices such as parsley, spring onions, basil, coriander, garlic, onions and others [53].<br>Add herbs, spices and other low salt flavourings, like no added salt tomato paste, to cooking in place of salt [51].

Table S1 (continued)

Food items	Examples of dietary behavior recommendations for salt reduction[a]
Citrus juice and vinegar (partly including recommendations on herbs and spices)	Use spices, savory vegetables, and sour fruits [19].<br>When cooking, replace or reduce the amount of salt you use. Alternatives could be garlic, citrus juice, salt-free seasonings, or spices [14].<br>Use flavorful ingredients. Onions, garlic, herbs, spices, citrus juices and vinegars can add flavor in place of some, or all, of the salt [15].<br>If you think it needs a boost of flavor, add freshly ground black pepper or a squeeze of fresh lemon or lime and taste it again before adding salt [15].<br>Try adding flavour to your food with herbs and spices, black pepper, garlic, chili or lemon juice instead of salt [18].<br>Boost flavor with herbs, spices, lemon, lime, vinegar, or salt-free seasoning blends instead of salt or salty seasonings like soy sauce, spice blends, or soup mixes [16].
Fruit	Eat more fruits and vegetables [14, 16].<br>Order vegetables with no salt added [14].
Vegetables	Eat more fruits and vegetables [14, 16].<br>Order fruit as a side item [14].

[a] Texts written in Japanese or Brazilian Portuguese were translated into English by the first author.

Table S2 Food codes in the analyzed meals that were used to generate each food type variable

**Variables**	**Food codes and food names**[a]
Staple food[b]	
Rice	[1001] Amaranth, whole grain, raw; [1002] Foxtail millet, milled grain, raw; [1003] Foxtail millet, glutinous cake; [1011] Proso millet, milled grain, raw; [1080] Rice, paddy rice, brown rice, raw; [1082] Rice, paddy rice, under-milled, raw; [1083] Rice, paddy rice, non-glutinous, well-milled, raw; [1084] Rice, short grain, paddy rice, well-milled, rice with embryo, raw; [1085] Rice, short grain, paddy rice, brown, "meshi" (cooked rice); [1086] Rice, short grain, paddy rice, half-milled, "meshi" (cooked rice); [1087] Rice, short grain, paddy rice, under-milled, "meshi" (cooked rice); [1088] Rice, short grain, paddy rice, nonglutinous rice, well-milled, "meshi" (cooked rice); [1089] Rice, short grain, paddy rice, well-milled, rice with embryo, "meshi" (cooked rice); [1090] Rice, short grain, paddy rice, brown, "zengayu" (gruel); [1092] Rice, short grain, paddy rice, under-milled, "zengayu" (gruel); [1093] Rice, short grain, paddy rice, well-milled, "zengayu" (gruel); [1097] Rice, short grain, paddy rice, well-milled, "gobugayu" (diluted gruel); [1102] Rice, short grain, upland rice, brown, raw; [1105] Rice, short grain, upland rice, well-milled, raw; [1106] Rice, short grain, upland rice, brown, "meshi" (cooked rice); [1108] Rice, short grain, upland rice, under-milled, "meshi" (cooked rice); [1109] Rice, short grain, upland rice, well-milled, "meshi" (cooked rice); [1110] Rice, non-glutinous rice products, quick-cooking rice, regular, raw; [1111] Rice, non-glutinous rice products, "Onigiri" (rice ball); [1112] Rice, non-glutinous rice products, "Yaki-onigiri" (baked rice ball); [1113] Rice, non-glutinous rice products, "Kiritanpo" (baked tube-shaped cooked rice); [1114] Rice, non-glutinous rice products, "Joshinko" (ordinary rice flour); [1117] Rice, glutinous rice products, rice cake; [1118] Rice, glutinous rice products, "Sekihan" (steamed rice with adzuki beans or cowpeas); [1126] Buckwheat, parboiled grain; [1151] Rice, paddy rice, glutinous rice, well-milled, raw ; [1151] Rice, paddy rice, glutinous rice, well-milled, raw; [1152] Rice, long grain, paddy rice, well-milled, raw; [1153] Rice, short grain, paddy rice, germinated brown rice, raw; [1154] Rice, short grain, paddy rice, glutinous rice, well-milled, "meshi" (cooked rice); [1155] Rice, short grain, paddy rice, germinated brown rice, "meshi" (cooked rice); [1156] Rice, non-glutinous rice products, quick-cooking rice, fortified product for school lunch, raw; [1168] Rice, long grain, paddy rice, well-milled, "meshi" (cooked rice); [15123] "Gohei-mochi" (rice cake, grilled with soy sauce, miso, sugar, and sesame paste)
Bread	[1026] Common wheat, bread, white; [1028] Common wheat, bread, white long roll; [1030] Common wheat, bread, hardtack; [1031] Common wheat, bread, French bread; [1032] Common wheat, bread, rye and wheat bread; [1033] Common wheat, bread, raisin bread; [1034] Common wheat, bread, soft rolls; [1035] Common wheat, bread, croissants, rich; [1036] Common wheat, bread, English muffins; [1037] Common wheat, bread, naan; [1148] Common wheat, bread, bagel; [1159] Rice, non-glutinous rice products, rice bread; [15069] Bun with filling, baked bun with strained bean paste filling, regular; [15070] Bun with filling, baked bun with custard cream filling, regular; [15071] Bun with filling, baked bun with strawberry jam filling; [15072] Bun with filling, "Korone" (horn-shaped bread), with chocolate cream filling; [15076] Cake and pastry, Danish pastry, Denmark-style, plain; [15125] Bun with filling, fried bun; [15126] Bun with filling, baked bun with strained bean paste filling, thin dough type; [15127] Bun with filling, fried bun with curry filling; [15128] Bun with filling, fried bun with curry filling, fried bun only; [15129] Bun with filling, fried bun with curry filling, curry filling only; [15130] Bun with filling, baked bun with custard cream filling, thin dough type; [15131] Bun with filling, baked bun with chocolate cream filling, thin dough type; [15132] Bun with filling, "Melon-pan" (sweet bun covered in a thin layer of crisp cookie dough)

Table S2 (continued)

Variables	Food codes and food names[a]
Noodles	[1039] Common wheat, "Udon" (thick wheat noodles), boiled; [1042] Common wheat, "Udon" (thick wheat noodles), dried noodles, boiled; [1044] Common wheat, "Somen and Hiyamugi" (thin wheat noodles), dried noodles, boiled; [1046] Common wheat, "Somen and Hiyamugi" (thin wheat noodles), dried noodles, hand-stretched, boiled; [1048] Common wheat, yellow alkaline noodles, boiled; [1049] Common wheat, yellow alkaline noodles, steamed noodles; [1051] Common wheat, yellow alkaline noodles, dried noodles, boiled; [1053] Common wheat, Okinawa noodles, boiled; [1056] Common wheat, instant Chinese noodles, dried by frying, seasoned; [1057] Common wheat, instant Chinese noodles, dried by frying, including attached seasonings, uncooked; [1058] Common wheat, instant Chinese noodles, dried without frying, including attached seasonings, uncooked; [1060] Common wheat, "Yakisoba", instant Chinese oil-fried noodles, dried by frying and in cups, including attached seasonings, uncooked; [1061] Common wheat, instant, Chinese noodles, dried without frying and packed in cups, including attached seasonings, uncooked; [1062] Common wheat, instant, "Udon" noodles, Japanese thick wheat noodles, dried by frying and packed in cups, including attached seasonings, uncooked; [1063] Durum wheat, macaroni and spaghetti, dry pasta, uncooked; [1064] Durum wheat, macaroni and spaghetti, dry pasta, boiled; [1115] Rice, non-glutinous rice products, dried noodles, raw; [1122] Buckwheat flour, straight; [1123] Buckwheat flour, inner layer; [1128] Buckwheat, fresh noodles, boiled; [1130] Buckwheat, dried noodles, boiled ; [1149] Durum wheat, macaroni and spaghetti, fresh pasta, uncooked; [1150] Common wheat, cold noodles, uncooked; [1160] Rice, non-glutinous rice products, fresh noodles, rice noodles, raw; [1191] Common wheat, instant Chinese oil-fried noodles, dried by frying and in cups, soy sauce flavor, including attached seasonings, uncooked; [2040] Starch products, "Harusame" (thin starch noodles), made from potato and sweet potato starches, dried, uncooked; [2061] Starch products, "Harusame" (thin starch noodles), made from mung bean starch, boiled; [2062] Starch products, "Harusame" (thin starch noodles), made from potato and sweet potato starches, boiled
Other staple foods	[1004] Common oats, oatmeal, raw; [1005] Barley, under-milled pressed grain, raw; [1006] Barley, pressed grain, raw; [1007] Barley, split grain, raw; [1010] Barley, roasted flour; [1013] Common wheat, whole grain, imported, soft, raw; [1015] Common wheat, soft flour, first grade; [1016] Common wheat, soft flour, second grade; [1018] Common wheat, medium-strength flour, first grade; [1019] Common wheat, medium-strength flour, second grade; [1020] Common wheat, hard flour, first grade; [1021] Common wheat, hard flour, second grade; [1023] Common wheat, hard flour, whole grain; [1024] Common wheat, premixed flour for pancake; [1076] Common wheat, pizza crust; [1120] Rice, glutinous rice products, "Shiratamako" (flour milled in water); [1121] Rice, glutinous rice products, "Domyojiko" (steamed flour); [1131] Corn, whole grain, yellow kernel, raw; [1132] Corn, Corn meal, yellow kernel, raw; [1133] Corn, Corn grits, yellow kernel, raw; [1134] Corn, Corn flour, yellow kernel, raw; [1137] Corn, cornflakes; [1138] Job's tears, milled grain, raw; [1142] Rye, whole flour; [1143] Rye, flour; [1146] Common wheat, premixed flour for "Okonomiyaki" (Japanese-style savory pancake with various ingredients); [1157] Rice, non-glutinous rice products, roasted brown rice flour; [1158] Rice, non-glutinous rice products, fine flour; [1161] Rice, bran; [1163] Corn, Corn meal, white kernel, raw; [1167] Quinoa, whole grain, raw; [1169] Rice, non-glutinous rice products, rice paper; [15034] Chinese style steamed bun, stuffed with strained bean paste; [15035] Chinese style steamed bun, stuffed with meat and vegetable; [15077] Cake and pastry, doughnuts, yeast-leavened, plain; [15078] Cake and pastry, doughnuts, cake-type, plain; [15083] Cake and pastry, thick pancake; [15084] Cake and pastry, waffles, with custard cream filling; [15093] Biscuits, crackers, oil-sprayed; [15094] Biscuits, crackers, soda
Soup	Not applicable (soups were identified based on dish names)

Table S2 (continued)

Variables	Food codes and food names[a]
Pickles	[6040] Turnip, pickles, salted pickles, leaves; [6041] Turnip, pickles, salted pickles, root with skin; [6042] Turnip, pickles, salted pickles, root without skin; [6043] Turnip, pickles, "Nukamiso-zuke" (pickled in salty rice bran paste), leaves; [6044] Turnip, pickles, "Nukamiso-zuke" (pickled in salty rice bran paste), root with skin; [6045] Turnip, pickles, "Nukamiso-zuke" (pickled in salty rice bran paste), root without skin; [6053] Leaf mustard, "Karashina", leaves, salted pickles; [6066] Cucumber, fruit, pickles, salted pickles; [6067] Cucumber, fruit, pickles, pickled in soy sauce; [6068] Cucumber, fruit, pickles, "Nukamiso-zuke" (pickled in salty rice bran paste); [6069] Cucumber, fruit, pickles, sweet type (pickled with seasoned vinegar); [6070] Cucumber, fruit, pickles, sour type (processed by lactic acid fermentation); [6074] Leaf green, "Mizuna", leaves, salted pickles; [6088] Stem mustard, pickles; [6104] Ginger, mature rhizome, pickles; [6105] Ginger, mature rhizome, pickles, sweetened; [6107] Oriental pickling melon, fruits, pickles, salted pickles; [6108] Oriental pickling melon, fruits, pickles, "Nara-zuke" (pickled with Sake lees); [6137] Japanese radishes, Daikon, root, pickles, "Nukamiso-zuke" (pickled in salty rice bran paste); [6138] Japanese radishes, Daikon, root, pickles, "Takuan-zuke" (pickled with rice bran and salt), made of salted Daikon; [6139] Japanese radishes, Daikon, root, pickles, "Takuan-zuke" (pickled with rice bran and salt), made of sun-dried Daikon; [6140] Japanese radishes, Daikon, root, pickles, "Moriguchi-zuke" (slender root cultivar pickled with Sake lees); [6141] Japanese radishes, Daikon, root, pickles, "Bettara-zuke" (pickled with rice koji); [6142] Japanese radishes, Daikon, root, pickles, "Miso-zuke" (pickled with miso); [6143] Japanese radishes, Daikon, root, pickles, "Fukujin-zuke" (pickled with Daikon, eggplant, immature sword pods and east Indian lotus rhizome); [6146] Chinese mustard, "Taisai", leaves, salted pickles; [6148] Leaf mustard, "Takana", leaves, salted pickles; [6195] Eggplant, pickles, salted pickles; [6196] Eggplant, pickles, ""Nukamiso-zuke"" (pickled with salty rice bran paste); [6197] Eggplant, pickles, "Koji-zuke" (pickled small oval type with rice koji); [6198] Eggplant, pickles, "Karashi-zuke" (pickled small oval type with mustard); [6199] Eggplant, pickles, "Shiba-zuke" (pickled with perilla leaf, cucumber and Myoga, etc.); [6230] Turnip green, "Nozawana", leaves, pickles, salted pickles; [6231] Turnip green, "Nozawana", leaves, pickles, seasoned; [6235] Chinese cabbage, head, pickles, salted pickles; [6236] Chinese cabbage, head, pickles, Kimchi ; [6242] Chayote, fruit, white-colored, salted pickles; [6253] Turnip, "Hinona", root with top portion, pickles, sweetened; [6255] Non-heading Chinese cabbage, "Hiroshimana", leaves, salted pickles; [6306] Japanese scallion, "Rakkyo", mature bulb, pickles, sweetened; [7020] Mume, salted pickles; [7021] Mume, pickles, seasoned; [7022] Mume, ""Umeboshi"" (pickled and dried mume), salted pickles ; [7023] Mume, ""Umeboshi"" (pickled and dried mume), seasoned pickles ; [7024] Mume,""Umebishio"" (sweetened puree of pickled mume) ; [7037] Olives, in brine, green; [7038] Olives, in brine, ripe; [7039] Olives, in brine, stuffed olives; [9023] Algae, kombu, "Tsukudani" (simmered in soy sauce and sugar); [9033] Algae, "Hitoegusa", "Tsukudani" (simmered in soy sauce and sugar)

Table S2 (continued)

Variables	Food codes and food names[a]
Fruit	[7003] Acerola, sour type, raw; [7006] Avocados, raw; [7007] Apricots, raw; [7008] Apricots, dried; [7009] Apricots, canned in heavy syrup; [7012] Strawberries, raw; [7015] Figs, raw; [7016] Figs, dried; [7017] Figs, canned in heavy syrup; [7018] Citrus, "Iyo", juice sacs; [7019] Mume, raw; [7026] Satsuma mandarins, segments, early ripening type, raw; [7027] Satsuma mandarins, segments, normal ripening type, raw; [7028] Satsuma mandarins, juice sacs, early ripening type, raw; [7029] Satsuma mandarins, juice sacs, normal ripening type, raw; [7035] Satsuma mandarins, canned in light syrup, solids; [7040] Oranges, navel, juice sacs, raw; [7041] Oranges, Valencia, imported from the U.S.A., juice sacs, raw; [7048] Oroblanco, juice sacs, raw; [7049] Japanese persimmons, non-astringent, raw ; [7050] Japanese persimmons, astringency removed, raw; [7051] Japanese persimmons, dried; [7053] Chinese quinces, raw; [7054] Kiwifruit, green flesh type, raw; [7056] Kumquats, whole, raw; [7057] Guava, red flesh type, raw; [7060] Gooseberries, raw; [7061] Oleasters, raw; [7062] Grapefruit, white flesh type, juice sacs, raw; [7067] Grapefruit, canned in light syrup; [7069] Carambola, raw; [7070] Sweet cherries, domestic, raw; [7071] Sweet cherries, imported from the U.S.A., raw; [7072] Sweet cherries, canned in heavy syrup; [7073] Pomegranates, raw; [7074] Citrus, "Sanbokan", juice sacs, raw; [7077] Watermelon, red flesh type, raw; [7078] Citrus, "Sudachi", peel, raw; [7080] Plums, Japanese plums, raw; [7081] Plums, European plums, raw; [7082] Plums, European plums, dried; [7085] Citrus, Seminole, juice sacs, raw; [7088] Pears, sand pears, raw; [7089] Pears, sand pears, canned in heavy syrup; [7090] Pears, Chinese white pears, raw; [7091] Pears, European pears, raw; [7092] Pears, European pears, canned in heavy syrup; [7093] Citrus, "Natsudaidai", juice sacs, raw; [7094] Citrus, "Natsudaidai", canned in heavy syrup; [7095] Jujube, dried; [7096] Dates, dried; [7097] Pineapple, raw; [7102] Pineapple, canned in heavy syrup; [7103] Pineapple, candied; [7104] Blue berried honeysuckle, raw; [7105] Citrus, "Hassaku", juice sacs, raw; [7106] Passion fruit, juice, fresh; [7107] Bananas, raw; [7108] Bananas, dried; [7109] Papaya, ripe, raw; [7110] Papaya, unripe, raw; [7111] Pitaya, raw; [7112] Citrus, "Hyuga-natsu", segments and albedo, raw; [7113] Citrus, "Hyuga-natsu", juice sacs, raw; [7114] Loquats, raw; [7115] Loquats, Raw, canned in heavy syrup; [7116] Grapes, without skin, raw; [7117] Grapes, raisins; [7122] Grapes, canned in heavy syrup; [7124] Blueberries, raw; [7126] Pummelo, juice sacs, raw; [7127] Pummelo, candied peel; [7129] Ponkan mandarins, juice sacs, raw; [7130] Oriental melon, yellow flesh type, raw; [7132] Mangoes, raw; [7134] Muskmelon, greenhouse culture, raw; [7135] Muskmelon, open culture, green flesh type, raw; [7136] Peaches, white flesh type, raw; [7138] Peaches, white flesh type, canned in heavy syrup, solids; [7139] Peaches, canned in heavy syrup, liquid; [7140] Nectarines, raw; [7141] Red bayberries, raw; [7142] Citrus, "Yuzu", peel, raw; [7144] Lychees, raw; [7146] Red raspberries, raw; [7147] Longans, dried; [7148] Apples, without skin, raw; [7153] Apples, canned in heavy syrup; [7155] Lemons, whole, raw; [7160] Strawberries, dried; [7161] Oranges, Fukuhara-orange, juice sacs, raw; [7162] Citrus, "Kawachi-bankan", juice sacs, raw; [7163] Citrus, "Kiyomi", juice sacs, raw; [7164] Grapefruit, red flesh type, juice sacs, raw; [7165] Citrus, "Shiranuhi", juice sacs, raw; [7166] Citrus, "Setoka", juice sacs, raw; [7167] Citrus, "Harumi", juice sacs, raw; [7168] Kiwifruit, yellow flesh type, raw; [7169] Guava, white flesh type, raw; [7170] Coconut, Nata de coco in syrup; [7171] Watermelon, yellow flesh type, raw; [7172] Blueberries, dried; [7173] Oriental melon, white flesh type, raw; [7174] Muskmelon, open culture, orange flesh type, raw; [7175] Peaches, yellow flesh type, canned in heavy syrup, solids; [7176] Apples, with skin, raw; [7178] Grapes, with skin, raw; [7179] Mangoes, dry; [7180] Apples, with skin, baked
Reduced-salt seasonings	[17086] Soy sauce, salt-reduced; [17119] Miso, salt-reduced

Table S2 (continued)

Variables	Food codes and food names[a]
Herbs and spices	[6153] Onions, bulb, raw; [6154] Onions, bulb, bleached in water; [6155] Onions, bulb, boiled; [6156] Onions, red onions, bulb, raw; [6223] Garlic, bulb, raw; [6224] Garlic, scape, raw; [6225] Garlic, scape, boiled; [6226] Welsh onions, "Nebuka-negi" (large variety, blanching cultivation), leaves, raw; [6227] Welsh onions, "Ha-negi" (large variety, green), leaves, raw; [6228] Welsh onions, "Konegi" (small variety), leaves, raw; [6336] Onions, bulb, sautéed; [6337] Onions, immature onions, bulb and stem, raw; [6349] Garlic, bulb, sautéed; [6350] Welsh onions, "Nebuka-negi" (large variety, blanching cultivation), leaves, boiled; [6351] Welsh onions, "Nebuka-negi" (large variety, blanching cultivation), leaves, sautéed; [6352] Welsh onions, "Ha-negi" (large variety, green), leaves, sautéed; [6365] Ginger, mature rhizome, raw, grated; [6366] Ginger, mature rhizome, raw, squeezed juice of grated ginger; [17055] Spices, allspice, ground; [17056] Spices, onion powder; [17057] Spices, mustard, powder; [17058] Spices, mustard, paste; [17059] Spices, mustard, yellow mustard; [17060] Spices, mustard, whole grain mustard; [17061] Spices, curry powder; [17062] Spices, clove, ground; [17063] Spices, pepper, black, ground; [17064] Spices, pepper, white, ground; [17065] Spices, pepper, black and white pepper mix, ground; [17066] Spices, "Sansho" (Japanese pepper), ground; [17067] Spices, cinnamon, ground; [17068] Spices, ginger, dried, ground; [17069] Spices, ginger, paste; [17070] Spice, sage, dried, ground; [17071] Spice, thyme, dried, ground; [17072] Spice, chili powder; [17073] Spice, red hot pepper, ground; [17074] Spice, nutmeg, ground; [17075] Spice, garlic, powder, without salt; [17076] Spice, garlic, paste; [17077] Spice, basil, dried, ground; [17078] Spices, parsley, dried; [17079] Spices, paprika; [17080] Spices, "Wasabi", powder, mixed with mustard powder; [17081] Spices, "Wasabi", paste; [17128] Spice, garlic, powder, with salt
Citrus juice and vinegar	[7052] Citrus, "Kabosu", juice, fresh; [7075] Citrus, "Shiikuwasha", juice, fresh; [7079] Citrus, "Sudachi", juice, fresh; [7083] Citrus, sour oranges, juice, fresh; [7143] Citrus, "Yuzu", juice, fresh; [7145] Limes, juice, fresh; [7156] Lemons, juice, fresh; [17015] Vinegar, grain vinegar; [17016] Vinegar, rice vinegar; [17017] Vinegar, fruit vinegar, wine vinegar; [17018] Vinegar, fruit vinegar, cider vinegar; [17090] Vinegar, black rice vinegar; [17091] Vinegar, fruit vinegar, Balsamic vinegar

Table S2 (continued)

Variables	Food codes and food names[a]
Moderately processed meat and seafood	[10006] Fish, horse mackerel, Japanese Jack mackerel, ""Hirakiboshi"" (salted and semi-dried split), raw; [10007] Fish, horse mackerel, Japanese Jack mackerel, ""Hirakiboshi"" (salted and semi-dried split), baked; [10013] Fish, horse mackerel, brownstriped mackerel scad, "Hirakiboshi" (salted and semi-dried split); [10035] Fish, Japanese sand lance, "Tsukudani" (simmered whole in soy sauce and sugar); [10036] Fish, Japanese sand lance, "Ameni" (simmered whole in glucose syrup and soy sauce); [10043] Fish, sardine, Pacific round herring, ""Maruboshi"" (salted and dried whole), raw; [10045] Fish, sardine, Japanese anchovy, "Niboshi" (boiled and dried whole); [10050] Fish, sardine, Japanese pilchard, "Shioiwashi" (salted pilchard), raw; [10051] Fish, sardine, Japanese pilchard, "Namaboshi" (mild salted and semi-dried whole), raw; [10052] Fish, sardine, Japanese pilchard, "Maruboshi" (salted and dried whole), raw; [10053] Fish, sardine, "Mezashi" (skewered, salted and semi-dried whole), raw; [10054] Fish, sardine, "Mezashi" (skewered, salted and semi-dried whole), baked; [10055] Fish, sardine, "Shirasuboshi" (boiled and dried whitebait), mild dried; [10056] Fish, sardine, "Shirasuboshi" (boiled and dried whitebait), semi-dried; [10058] Fish, sardine, Japanese anchovy, "Mirinboshi" (split seasoned with Mirin and dried); [10060] Fish, sardine, Japanese pilchard, canned products, in brine; [10061] Fish, sardine, Japanese pilchard, canned products, with seasoning; [10063] Fish, sardine, Japanese pilchard, canned products, in oil; [10064] Fish, sardine, Japanese pilchard, "Kabayaki" (baked and seasoned fillet), canned products; [10070] Fish, eel, "Kabayaki" (seasoned and baked fillet); [10072] Fish, black scraper, "Ajitsuke-hirakiboshi" (seasoned and dried fillet) ; [10093] Fish, skipjack tuna and frigate mackerel, processed products, "Kezuri-bushi" (shaved "Katsuo-bushi"), simmered in soy sauce and sugar (cf. 10091"Katsuo-bushi"); [10094] Fish, skipjack tuna and frigate mackerel, processed products, "Kakuni" (meat cube boiled in soy sauce and sugar); [10096] Fish, skipjack tuna and frigate mackerel, canned products, flaked meat with seasoning; [10097] Fish, skipjack tuna and frigate mackerel, canned products, flaked meat in oil; [10106] Fish, righteye flounder, dried; [10112] Fish, blue sprat, seasoned and dried; [10125] Fish, dotted gizzard shad, "Amazu-zuke" (marinated in vinegar and sugar); [10128] Fish, salmon and trout, pink salmon, salted; [10129] Fish, salmon and trout, pink salmon, canned in brine; [10137] Fish, salmon and trout, chum salmon, "Aramaki" (salted whole body), raw; [10138] Fish, salmon and trout, chum salmon, "Aramaki" (salted whole body), baked; [10139] Fish, salmon and trout, chum salmon, "Shiozake" (salted salmon), raw; [10140] Fish, salmon and trout, chum salmon, "Ikura" (salted roe) ; [10141] Fish, salmon and trout, chum salmon, "Sujiko" (salted ovary); [10142] Fish, salmon and trout, chum salmon, "Mefun" (salted and fermented kidney); [10143] Fish, salmon and trout, chum salmon, canned in brine; [10151] Fish, salmon and trout, sockeye salmon, smoked; [10161] Fish, mackerel, processed products, "Shiosaba" (plain salted fillet) ; [10162] Fish, mackerel, processed products, "Hirakiboshi" (mild salted and semi-dried split) ; [10163] Fish, mackerels, processed products, "Shimesaba" (vinegar marinated fillet); [10164] Fish, mackerel, canned products, boiled in brine; [10165] Fish, mackerel, canned products, boiled with miso; [10166] Fish, mackerel, canned products, boiled with seasoning; [10175] Fish, Pacific saury, "Hirakiboshi" (mild salted and semi-dried split); [10176] Fish, Pacific saury, "Mirinboshi" (seasoned with Mirin and dried fillet); [10177] Fish, Pacific saury, canned products, boiled with seasoning; [10178] Fish, Pacific saury, canned products, "Kabayaki" (baked and seasoned fillet); [10180] Fish, Shishamo smelt, semi-dried, raw; [10181] Fish, Shishamo smelt, semi-dried, baked; [10182] Fish, Atlantic capelin, semi-dried, raw; [10183] Fish, Atlantic capelin, semi-dried, baked; [10194] Fish, sea bream, red sea bream, cultured, with integument, boiled; [10201] Fish, cod, walleye pollock, ""Sukimidara"" (skinned, salted and dried fillet); [10202] Fish, cod, walleye pollock, "Tarako" (salted roe), raw ; [10203] Fish, cod, walleye pollock, "Tarako" (salted roe), baked ; [10204] Fish, cod, walleye pollock, "Karashi-mentaiko" (salted roe with red hot pepper powder) ; [10208] Fish, cod, Pacific cod, salted fillet; [10209] Fish, cod, Pacific cod, dried split; [10210] Fish, cod, Pacific cod, "Denbu" (mashed and seasoned meat); [10220] Fish, Pacific herring, "Hirakiboshi" (dried split); [10221] Fish, Pacific herring, smoked; [10224] Fish, Pacific herring, roe, salt-cured products, desalted; [10227] Fish, yellowfin goby, "Kanroni" (simmered whole in soy sauce and sugar) ; [10229] Fish, sailfin sandfish, "Namaboshi" (salted and semi-dried whole) ; [10247] Fish, Atka mackerel, salted; [10248] Fish, Atka mackerel, ""Hirakiboshi"" (mild salted and semi-dried split), raw; [10250] Fish, striped mullet, "Karasumi" (salted and dried roe); [10260] Fish, tuna, canned products, flaked light meat in brine; [10261] Fish, tuna, canned products, flaked white meat in brine; [10262] Fish, tuna, canned products, flaked meat with seasoning; [10263] Fish, tuna, canned products, flaked light meat in oil; [10264] Fish, tuna, canned products, flaked white meat in oil; [10277] Fish, Japanese smelt, ""Tsukudani""

Table S2 (continued)

Variables	Food codes and food names[a]
Moderately processed meat and seafood	(simmered whole in soy sauce and sugar); [10278] Fish, Japanese smelt, ""Ameni"" (simmered whole in glucose syrup and soy sauce); [10282] Mollusks, short-necked clam, ""Tsukudani"" (simmered meat in soy sauce and sugar); [10283] Mollusks, short-necked clam, canned products, boiled in brine; [10284] Mollusks, short-necked clam, canned products, boiled with seasoning; [10288] Mollusks, abalone, canned in brine; [10291] Mollusks, Escargot Apple snails, canned in brine; [10294] Mollusks, Pacific oyster, canned in oil, smoked; [10314] Mollusks, giant ezo-scallop, adductor muscle, "Niboshi" (boiled and dried) ; [10315] Mollusks, giant ezo-scallop, adductor muscle, canned products, boiled in brine; [10326] Crustacean, Sakura shrimp, "Niboshi" (boiled and dried); [10331] Crustacean, processed shrimp, "Tsukudani" (simmered whole in soy sauce and sugar); [10337] Crustacean, snow crab, canned products, boiled in brine; [10340] Crustacean, red king crab, canned products, boiled in brine; [10341] Crustacean, processed crab, "Ganzuke" (salted and fermented fiddler crabs); [10350] Mollusks, firefly squid, seasoned and smoked; [10351] Mollusks, firefly squid, "Tsukudani" (simmered whole in soy sauce and sugar); [10353] Mollusks, processed squid, "Surume" (dried squid); [10354] Mollusks, processed squid, "Saki-ika" (dried, seasoned and shredded squid); [10355] Mollusks, processed squid, seasoned and smoked; [10356] Mollusks, processed squid, "Kiriika-ameni" (dried shredded squid, simmered in glucose syrup and soy sauce); [10357] Mollusks, processed squid, "Ika-arare" (dried squid flakes, simmered in glucose syrup and soy sauce); [10358] Mollusks, processed squid, "Shiokara" (salted and fermented meat and liver); [10359] Mollusks, processed squid, canned with seasoning; [10363] Crustacean, opossum shrimp, ""Tsukudani"" (simmered whole in soy sauce and sugar); [10364] Crustacean, opossum shrimp, ""Shiokara"" (salted and fermented); [10366] Sea urchin, "Tsubu-uni" (salted whole gonads); [10367] Sea urchin, "Neri-uni" (salted whole gonad paste); [10369] Crustacean, Antarctic krill, boiled; [10370] Jellyfish, Salted, desalted; [10375] Sea squirt, ""Shiokara"" (salted and fermented meat and viscera); [10397] Fish, sardine, anchovy, canned products, in oil; [10412] Fish, Atka mackerel, ""Hirakiboshi"" (mild salted and semi-dried split), baked; [10421] Fish, flying fish, "Niboshi" (boiled and dried whole); [10422] Fish, flying fish, "Yakiboshi" (baked and dried whole); [11104] Beef products, roast beef; [11105] Beef products, corned beef, canned; [11106] Beef products, canned with seasoning; [11108] Beef products, smoked tongue; [11174] Pork, ham, bone-in; [11175] Pork, ham, boneless; [11176] Pork, ham, loin; [11177] Pork, ham, shoulder; [11181] Pork, ham, uncooked ham, fresh; [11182] Pork, ham, uncooked ham, ripened; [11183] Pork, bacon; [11184] Pork, bacon loin; [11185] Pork, shoulder bacon; [11195] Pork, roast pork; [11197] Pork, smoked liver; [11237] Chicken, canned products, roast meat with seasoning; [11241] Rice hopper, Tsukudani (simmered whole without wings and legs in soy sauce and sugar)

Table S2 (continued)

Variables	Food codes and food names[a]
Highly processed meat and seafood	[10376] Surimi products, "Kanifumi-kamaboko" (imitation crab meat made from surimi) ; [10377] Surimi products, "Kobumaki-kamaboko" (surimi rolled in kombu, steamed); [10378] Surimi products, "Sumaki-kamaboko" (steamed kamaboko covered with straw); [10379] Surimi products, "Mushi-kamaboko" (steamed kamaboko); [10380] Surimi products, "Yakinuki-kamaboko" (baked kamaboko); [10381] Surimi products, "Yaki-chikuwa" (baked tubular kamaboko); [10382] Surimi products, "Datemaki" (sweet rolled omelet made of egg and surimi); [10383] Surimi products, "Tsumire" (boiled red meat fish paste) ; [10384] Surimi products, "Naruto" (boiled stick kamaboko with cross section of red swirl); [10385] Surimi products, "Hanpen" (boiled fishcake made of surimi, yam paste and starch); [10386] Surimi products, "Satsuma-age" (fried surimi); [10387] Surimi products, fish ham; [10388] Surimi products, fish sausage; [10390] Fish, horse mackerel, Japanese Jack mackerel, with integument, breaded and fried; [10392] Fish, horse mackerel, Japanese Jack mackerel, small fish, with bones, floured and deep-fried; [10395] Fish, sardine, Japanese pilchard, breaded and fried; [10400] Fish, Japanese whiting, tempura; [10403] Fish, mackerel, chub mackerel, breaded and fried; [10409] Fish, cod, walleye pollock, breaded and fried; [10416] Crustacean, whiteleg shrimp, tempura; [10419] Mollusks, Japanese common squid, mantle, tempura; [10423] Surimi products, "Kuro-hanpen" (boiled black fishcake made of surimi and starch); [11178] Pork, ham, pressed; [11179] Mutton, ham, pressed, mixed with fish meat; [11180] Pork, ham, chopped; [11186] Pork, sausage, Vienna; [11187] Pork, sausage, semi-dry; [11188] Pork, sausage, dry; [11189] Pork, sausage, Frankfurter; [11190] Pork, sausage, Bologna; [11191] Pork, sausage, Lyoner; [11192] Pork, sausage, liver; [11193] Pork, sausage, mixed with fish meat; [11194] Pork, sausage, fresh sausage; [11196] Pork, liver paste; [11276] Pork, large type breed, loin, lean and fat, "Tonkatsu" (breaded and fried pork); [11279] Pork, large type breed, fillet, lean, "Tonkatsu" (breaded and fried pork); [11289] Chicken, broiler, thigh, meat with skin, "Karaage"(floured and deep-fried chicken); [11290] Chicken, broiler, thigh, meat without skin, "Karaage"(floured and deep-fried chicken); [11292] Chicken, nuggets; [11293] Chicken, "Tsukune" (Japanese chicken meatball)
Alcoholic beverages	[16001] Fermented alcoholic beverage, "Sake", regular; [16002] Fermented alcoholic beverage, "Sake", "Junmai" (made with rice, water, and koji only); [16003] Fermented alcoholic beverage, "Sake", "Honjozo" (made with rice, water, koji and distilled alcohol. The rice used must be polished to at least 70%); [16004] Fermented alcoholic beverage, "Sake", "Ginjo" (brewed with labor-intensive steps, using highly polished rice); [16005] Fermented alcoholic beverage, "Sake", "Junmai-ginjo" (made with rice, water, and koji only, brewed with labor-intensive steps, using highly polished rice); [16006] Fermented alcoholic beverage, beer, pale; [16007] Fermented alcoholic beverage, beer, black; [16008] Fermented alcoholic beverage, beer, stout; [16009] Fermented alcoholic beverage, "Happoshu" (beer-like beverage with less than 67% malt content); [16010] Fermented alcoholic beverage, wine, white; [16011] Fermented alcoholic beverage, wine, red; [16012] Fermented alcoholic beverage, wine, rose; [16013] Fermented alcoholic beverage, Shaoxing wine; [16014] Distilled alcoholic beverage, "Shochu", distilled through a continuous still; [16015] Distilled alcoholic beverage, "Shochu", distilled through a pot still; [16016] Distilled alcoholic beverage, whisky; [16017] Distilled alcoholic beverage, brandy; [16018] Distilled alcoholic beverage, vodka; [16019] Distilled alcoholic beverage, gin; [16020] Distilled alcoholic beverage, rum; [16022] Compound alcoholic beverage, "Umeshu" (plum liquor made from Japanese apricots); [16027] Compound alcoholic beverage, medicinal liqueur; [16028] Compound alcoholic beverage, curacao; [16029] Compound alcoholic beverage, fortified wine, sweet type; [16031] Compound alcoholic beverage, vermouth, sweet type; [16059] Compound alcoholic beverage, "Can chu-hi" (Cocktail in a can), lemon flavor

Table S2 (continued)

Variables	Food codes and food names[a]
Salt-based seasonings	[17001] Japanese Worcester sauce, common type; [17002] Japanese Worcester sauce, semi-thick type; [17003] Japanese Worcester sauce, thick type; [17004] Hot seasoning, Doubanjiang; [17005] Hot seasoning, hot pepper sauce; [17007] Soy sauce, "Koikuchi-shoyu" (common soy sauce); [17008] Soy sauce, "Usukuchi-shoyu" (light color soy sauce); [17009] Soy sauce, "Tamari-shoyu" (full-bodied soy sauce); [17010] Soy sauce, "Saishikomi-shoyu" (refermented soy sauce); [17011] Soy sauce, "Shiro-shoyu" (extra light color soy sauce); [17012] Edible salt, common salt, sodium chloride ≥ 99%; [17013] Edible salt, common salt, sodium chloride ≥ 95%; [17014] Edible salt, refined salt, sodium chloride ≥ 99.5%, containing magnesium carbonate; [17027] Stock cubes, meat and vegetable; [17028] Stock powder, "Katsuo-bushi" (cf. 10091 "Katsuo-bushi") ; [17029] Japanese noodle soup, non-concentrated (soy sauce base); [17030] Japanese noodle soup, triple-concentrated (soy sauce base); [17031] Seasoning sauce, oyster sauce; [17032] Seasoning sauce, Mapo tofu sauce; [17033] Seasoning sauce, meat sauce; [17036] Tomato products, ketchup; [17037] Tomato products, tomato sauce; [17038] Tomato products, chili sauce; [17039] Dressing, soy sauce based, fat-free; [17040] Dressing, French dressing; [17041] Dressing, thousand island dressing; [17042] Dressing, mayonnaise, whole egg type; [17043] Dressing, mayonnaise, egg yolk type; [17044] Miso, rice-koji miso, sweet type; [17045] Miso, rice-koji miso, light yellow type; [17046] Miso, rice-koji miso, red type; [17047] Miso, barley-koji miso; [17048] Miso, soybean-koji miso; [17049] Miso, instant miso soup, powdered type; [17050] Miso, instant miso soup, Paste type; [17051] Roux, Japanese curry roux, instant; [17052] Roux, hash and rice roux, instant; [17085] Japanese Worcester sauce, sweet thick type for "Okonomiyaki" (Japanese-style savory pancake with various ingredients); [17087] Soy sauce, pre-seasoned with soup stock; [17088] Soy sauce, soy glazed; [17089] Edible salt, refined salt, sodium chloride ≥ 99.5%, not containing magnesium carbonate; [17092] Stock powder, for "Oden" (Japanese winter hodgepodge); [17093] Stock powder, chicken, pork and vegetable; [17094] Seasoning sauce, sweet vinegar; [17095] Seasoning sauce, sauce for "Ebichiri" (shrimp with chili sauce); [17097] Seasoning sauce, "Gomasu" (vinegar mixture containing sesami, soy sauce and sugar); [17098] Seasoning sauce, sesame sauce; [17099] Seasoning sauce, "Sanbaizu" (vinegar mixture containing sesami, soy sauce and mirin); [17100] Seasoning sauce "Nihaizu" (vinegar mixture containing soy sauce); [17101] Seasoning sauce, sweetened vinegar for "Inarizushi" (fried tofu pouch filled with sushi rice); [17102] Seasoning sauce, sweetened vinegar for "Nigirizushi" (hand-pressed sushi); [17103] Seasoning sauce, sweetened vinegar for "Makizushi" (rolled sushi) and "Hakozushi"(pressed sushi); [17104] Seasoning sauce, Chinese style vinegar; [17105] Seasoning sauce, demi-glace sauce; [17106] Seasoning sauce, Tian Mian Jiang (sweet soybean paste); [17107] Seasoning sauce, Nam pla (fish sauce); [17108] Seasoning sauce, for "Hiyashi-chuka" (ramen noodles in a cold sweet soy sauce broth topped with meat and vegetables); [17109] Seasoning sauce, white sauce; [17110] Seasoning sauce, ponzu vinegar with soy sauce; [17111] Seasoning sauce, marinade; [17112] Seasoning sauce, for "Yakitori" (grilled chicken skewers); [17113] Seasoning sauce, barbecue sauce, soy sauce based; [17114] Seasoning sauce, for "Mitarashi" (skewed rice dumplings in a sweet soy glaze); [17115] Seasoning sauce, "Yuzu kosho" (spicy paste made from chili, yuzu zest and salt); [17116] Dressing, soy sauce based, with oil; [17117] Dressing, sesame dressing; [17118] Dressing, mayonnaise-type, low calorie type; [17120] Miso, pre-seasoned with soup stock; [17121] Miso, "Karashi-miso" (miso sauce containing mustard, vinegar and sugar); [17122] Miso, "Goma-miso" (miso sauce containing sesame); [17123] Miso, "Su-miso" (miso sauce containing vinegar and mustard); [17124] Miso, "Neri-miso" (miso sauce containing egg and mirin); [17125] Seasoning mix for "Ochazuke" (bowl of rice soaked in dashi broth); [17126] Instant soup mix, "Sumashi-jiru" (Japanese traditional clear-soup); [17127] "Furikake" (Seasoning mix for rice, containing dried seaweed and egg); [17133] Seasoning sauce, "Ikanago-shoyu" (fish sauce made from japanese sand lance); [17134] Seasoning sauce, "Ishiru" or "Ishiri" (fish sauce made from fish or squid-guts); [17135] Seasoning sauce, "Shottsuru" (fish sauce made from sandfish and other small fish); [17136] seasoning sauce for kimchi; [17137] Seasoning sauce, ponzu vinegar with soy sauce, commercial; [17138] Cooking sake

Table S2 (continued)

Variables	Food codes and food names[a]
Vegetables[c]	[6003] Chive, "Asatsuki", leaves, raw; [6004] Chive, "Asatsuki", leaves, boiled; [6005] Angelica, "Ashitaba", stems and leaves, raw; [6006] Angelica, "Ashitaba", stems and leaves, boiled; [6007] Asparagus, green, shoots, raw; [6008] Asparagus, green, shoots, boiled; [6009] Asparagus, white, canned in brine; [6010] Kidney beans, "Sayaingen", immature pods, raw; [6011] Kidney beans, "Sayaingen", immature pods, boiled; [6012] Japanese spikenard, "Udo", blanching cultivation, stem, raw; [6013] Japanese spikenard, "Udo", blanching cultivation, stem, bleached in water; [6014] Japanese spikenard, "Udo", outdoor cultivation, stem, raw; [6015] Soybeans, immature, raw; [6016] Soybeans, immature, boiled ; [6017] Soybeans, immature, frozen; [6018] Endive, leaves, raw; [6019] Peas, stem and leaves, raw; [6020] Peas, snow peas, immature pods, raw; [6021] Peas, snow peas, immature pods, boiled; [6022] Peas, snap peas, immature pods, raw; [6023] Peas, green peas, raw; [6024] Peas, green peas, boiled; [6025] Peas, green peas, frozen; [6026] Peas, green peas, canned in brine; [6027] Non-heading Chinese cabbage, "Osaka-shirona", leaves, raw; [6028] Non-heading Chinese cabbage, "Osaka-shirona", leaves, boiled; [6030] Japanese saltwort, stems and leaves, raw; [6031] Japanese saltwort, stems and leaves, boiled; [6032] Okra, pods, raw; [6033] Okra, pods, boiled; [6034] Turnip, leaves, raw; [6035] Turnip, leaves, boiled; [6036] Turnip, root, with skin, raw; [6037] Turnip, root, with skin, boiled; [6038] Turnip, root, without skin, raw; [6039] Turnip, root, without skin, boiled; [6046] Pumpkin and squash, Japanese squash, fruit, raw; [6047] Pumpkin and squash, Japanese squash, fruit, boiled; [6048] Pumpkin and squash, winter squash, fruit, raw; [6049] Pumpkin and squash, winter squash, fruit, boiled ; [6050] Pumpkin and squash, winter squash, fruit, frozen; [6051] Pumpkin and squash, spaghetti squash, fruit, raw; [6052] Leaf mustard, "Karashina", leaves, raw; [6054] Cauliflower, inflorescence, raw; [6055] Cauliflower, inflorescence, boiled; [6056] Gourd, "Kanpyo" (dried gourd strips), raw; [6057] Gourd, "Kanpyo" (dried gourd strips), boiled; [6058] Chrysanthemum, petals, raw; [6059] Chrysanthemum, petals, boiled; [6060] Chrysanthemum, "Kikunori" (sheet of dried chrysanthemum petals); [6061] Cabbage, common, head, raw; [6062] Cabbage, common, head, boiled; [6063] Cabbage, green ball, head raw; [6064] Cabbage, red cabbage, head raw; [6065] Cucumber, fruit, raw; [6071] Japanese victory onion, leaves, raw; [6072] Leaf green, "Mizuna", leaves, raw; [6073] Leaf green, "Mizuna", leaves, boiled; [6075] Leaf celery, stems and leaves, raw; [6077] Watercress, stems and leaves, raw; [6078] Arrowhead, tuber, raw; [6079] Arrowhead, tuber, boiled; [6080] Kale, leaves, raw; [6082] Kohlrabi, enlarged stems, boiled; [6083] Ostrich-feather fern, fiddlehead, raw; [6084] Edible burdock, root, raw; [6085] Edible burdock, root, boiled; [6086] Spinach mustard, "Komatsuna", leaves, raw; [6087] Spinach mustard, "Komatsuna", leaves, boiled; [6089] Non-heading Chinese cabbage, "Santosai", leaves, raw; [6090] Non-heading Chinese cabbage, "Santosai", leaves, boiled; [6092] Winged beans, immature pods, raw; [6093] Sweet peppers, "Shishito", fruit, raw; [6094] Sweet peppers, "Shishito", fruit, sautéed; [6095] Perilla, "Shiso", leaves, raw; [6096] Perilla, "Shiso", immature seeds, raw; [6097] Yandlong beans, immature pods, raw; [6098] Yandlong beans, immature pods, boiled; [6099] Garland chrysanthemum, leaves, raw; [6100] Garland chrysanthemum, leaves, boiled; [6102] Ginger, immature rhizome with stem, raw; [6103] Ginger, mature rhizome, raw; [6106] Oriental pickling melon, fruit, raw; [6109] Taro, petiole, fresh, raw; [6110] Taro, petiole, fresh, boiled; [6111] Taro, petiole, dried, raw; [6112] Taro, petiole, dried and boiled; [6115] Turnip, "Sugukina", leaves and root, pickles; [6116] Zucchini, fruit, raw; [6117] Water dropwort, stems and leaves, raw; [6118] Water dropwort, stems and leaves, boiled; [6119] Celery, petiole, raw; [6120] Japanese royal fern, fiddleheads, fresh, raw; [6121] Japanese royal fern, fiddleheads, fresh, boiled; [6122] Japanese royal fern, fiddleheads, dried, raw; [6123] Japanese royal fern, fiddleheads, dried, boiled; [6124] Broad beans, immature beans, raw; [6125] Broad beans, immature beans, boiled; [6126] Tatsoi, leaves, raw; [6128] Japanese radishes, Daikon, sprouts, raw; [6129] Japanese radishes, Daikon, cultivar for leaf use, leaves, raw; [6130] Japanese radishes, Daikon, leaves, raw; [6131] Japanese radishes, Daikon, leaves, boiled; [6132] Japanese radishes, Daikon, root with skin, raw; [6133] Japanese radishes, Daikon, root with skin, boiled; [6134] Japanese radishes, Daikon, root without skin, raw; [6135] Japanese radishes, Daikon, root without skin, boiled; [6136] Japanese radishes, Daikon, "Kiriboshi-daikon" (cut and dried Daikon root), raw; [6144] Chinese mustard, "Taisai", young leaves, raw; [6145] Chinese mustard, "Taisai", leaves, raw ; [6147] Leaf mustard, "Takana", leaves, raw; [6149] Bamboo shoots, raw; [6150] Bamboo shoots, boiled; [6151] Bamboo shoots, canned in water; [6152] Bamboo shoots, "Shinachiku" (boiled, fermented and salted bamboo shoots), desalted; [6157] Japanese angelica-tree, spears, raw; [6158] Japanese angelica-tree, spears, boiled; [6159] Chicory, spears, raw; [6160] Green bok choy, leaves, raw; [6161] Green bok choy,

Table S2 (continued)

Variables	Food codes and food names[a]
Vegetables[c]	leaves, boiled; [6164] New Zealand spinach, stems and leaves, raw; [6165] Malabar nightshade, stems and leaves, raw; [6166] Malabar nightshade, stems and leaves, boiled; [6168] Leopard plant, petiole, boiled; [6169] Hot peppers, leaves and fruits, raw; [6170] Hot peppers, leaves and fruits, sautéed; [6171] Hot peppers, fruit, raw; [6172] Hot peppers, fruit, dried; [6173] Chinese preserving melon, fruit, raw; [6174] Chinese preserving melon, fruit, boiled; [6175] Corn, sweet corn, immature kernels, raw; [6176] Corn, sweet corn, immature kernels, boiled; [6177] Corn, sweet corn, immature kernels on cob, frozen; [6178] Corn, sweet corn, immature kernels, frozen; [6179] Corn, sweet corn, canned products, cream style; [6180] Corn, sweet corn, canned products, whole kernel style; [6181] Corn, sweet corn, young ear, raw; [6182] Tomatoes, fruit, red, raw; [6183] Tomatoes, cherry tomatoes, fruit, red, raw; [6184] Tomatoes, canned products, whole, without salt; [6187] Red chicory, leaves, raw; [6189] Semi-heading Chinese cabbage, "Nagasaki-hakusai", leaves, raw; [6190] Semi-heading Chinese cabbage, "Nagasaki-hakusai", leaves, boiled; [6191] Eggplant, Japanese type, fruit, raw; [6192] Eggplant, Japanese type, fruit, boiled; [6193] Eggplant, western type, fruit, raw; [6194] Eggplant, western type, fruit, deep-fried; [6201] Turnip rape, flower buds and stems, raw; [6202] Turnip rape, flower buds and stems, boiled; [6203] Rape, stems and leaves, raw; [6204] Rape, stems and leaves, boiled; [6205] Bitter melon, fruit, raw; [6206] Bitter melon, fruit, sautéed; [6207] Chinese chive, leaves, raw; [6208] Chinese chive, leaves, boiled; [6209] Chinese chive, scape and flower bud, raw; [6211] Carrots, cultivar for leaf use, leaves, raw; [6212] Carrot, regular (European type), root with skin, raw; [6213] Carrot, regular (European type), root with skin, boiled; [6214] Carrot, regular (European type), root without skin, raw; [6215] Carrot, regular (European type), root without skin, boiled; [6216] Carrot, regular (European type), root, frozen; [6218] Carrot, "Kintoki" (oriental type), root with skin, raw; [6220] Carrot, "Kintoki" (oriental type), root without skin, raw; [6222] Carrot, baby carrot, root, raw; [6229] Turnip green, "Nozawana", leaves, raw; [6232] Wild onion, "Nobiru", bulb and leaves, raw; [6233] Chinese cabbage, head, raw; [6234] Chinese cabbage, head, boiled; [6237] Bok choy, leaves, raw; [6238] Basil, leaves, raw; [6239] Parsley, leaves, raw; [6240] Little radish, root, raw; [6241] Chayote, fruit, white-colored, raw; [6243] Red beet, root, raw ; [6244] Red beet, root, boiled ; [6245] Sweet peppers, fruit, green, raw; [6246] Sweet peppers, fruit, green, sautéed; [6247] Sweet peppers, fruit, red, raw; [6248] Sweet peppers, fruit, red, sautéed; [6249] Sweet peppers, fruit, yellow, raw; [6250] Sweet peppers, fruit, yellow, sautéed; [6252] Turnip, "Hinona", root with top portion, raw; [6254] Non-heading Chinese cabbage, "Hiroshimana", leaves, raw; [6256] Japanese butterbur, petiole, raw; [6257] Japanese butterbur, petiole, boiled; [6258] Japanese butterbur, inflorescence, raw; [6259] Japanese butterbur, inflorescence, boiled; [6260] Hyacinth beans, immature pods, raw; [6261] Swiss chard, leaves, raw; [6262] Swiss chard, leaves, boiled; [6263] Broccoli, inflorescence, raw; [6264] Broccoli, inflorescence, boiled; [6265] Sponge gourd, immature fruit, raw; [6266] Sponge gourd, immature fruit, boiled; [6267] Spinach, leaves, all season, raw; [6268] Spinach, leaves, all season, boiled; [6269] Spinach, leaves, frozen; [6270] Horseradish, rhizome, raw; [6272] Turnip green, "Mizukakena", leaves, raw; [6274] Japanese hornwort, "Kiri-mitsuba" (branched in a dark place), leaves, raw; [6275] Japanese hornwort, "Kiri-mitsuba" (branched in a dark place), leaves, boiled; [6276] Japanese hornwort, "Ne-mitsuba" (branched by covering with soil), leaves, raw; [6277] Japanese hornwort, "Ne-mitsuba" (branched by covering with soil), leaves, boiled; [6278] Japanese hornwort, "Ito-mitsuba" (young plants), leaves, raw; [6279] Japanese hornwort, "Ito-mitsuba" (young plants), leaves, boiled; [6280] Japanese ginger, "Myoga", spike, raw; [6281] Japanese ginger, "Myoga", young stems and leaves, raw; [6282] Yam, bulbil, raw; [6283] Brussels sprouts, head, raw; [6284] Brussels sprouts, head, boiled; [6285] Water pepper sprouts, raw; [6286] Bean sprouts, alfalfa sprouts, raw; [6287] Bean sprouts, soybean sprouts, raw; [6288] Bean sprouts, soybean sprouts, boiled; [6289] Bean sprouts, black gram sprouts, raw; [6290] Bean sprouts, black gram sprouts, boiled; [6291] Bean sprouts, mung bean sprouts, raw; [6292] Bean sprouts, mung bean sprouts, boiled; [6293] Nalta jute, stems and leaves, raw ; [6294] Nalta jute, stems and leaves, boiled ; [6295] Thistle root, "Yamagobo", pickled with miso; [6296] Lily, bulb, raw; [6297] Lily, bulb, boiled; [6298] Water convolvulus, stems and leaves, raw; [6299] Water convolvulus, stems and leaves, boiled; [6301] Japanese wormwood, leaves, raw; [6302] Japanese wormwood, leaves, boiled; [6304] Peanuts, immature beans, boiled; [6305] Japanese scallion, "Rakkyo", mature bulb, raw; [6307] Japanese scallion, "Rakkyo", immature bulb, raw; [6310] Garden rhubarb, petiole, raw; [6311] Garden rhubarb, petiole, boiled; [6312] Lettuce, head lettuce, crisp type, soil culture, head, raw; [6313] Lettuce, head lettuce, butter type, leaves, raw; [6314] Lettuce, green leaf lettuce, leaves, raw; [6315] Lettuce, red leaf lettuce, leaves, raw; [6316] Lettuce, Romaine

Table S2 (continued)

Variables	Food codes and food names[a]
Vegetables[c]	lettuce, leaves, raw; [6317] East Indian lotus root, rhizome, raw; [6318] East Indian lotus root, rhizome, boiled; [6319] Rocket salad, leaves, raw; [6320] Green onion, "Wakegi", leaves, raw; [6321] Green onion, "Wakegi", leaves, boiled; [6322] Wasabi, rhizome, raw; [6323] Wasabi, pickled with Sake lees; [6324] Bracken fern, fiddleheads, raw; [6325] Bracken fern, fiddleheads, boiled; [6326] Bracken fern, fiddleheads, dried, raw; [6327] Asparagus, green, shoots, sautéed; [6328] Aloe, leaves, raw; [6329] Peas, sprouts, raw; [6330] Peas, sprouts, boiled; [6331] Peas, sprouts, sautéed; [6332] Pumpkin and squash, winter squash, fruit, baked; [6333] Cabbage, common, head, sautéed; [6334] Japanese radishes, Daikon, "Kiriboshi-daikon" (cut and dried Daikon root), rehydrated and boiled; [6335] Japanese radishes, Daikon, "Kiriboshi-daikon" (cut and dried Daikon root), rehydrated and sautéed; [6338] Green bok choy, leaves, sautéed; [6339] Corn, sweet corn, immature kernels, microwaved; [6342] Eggplant, Japanese type, fruit, sautéed; [6343] Eggplant, Japanese type, fruit, tempura; [6344] Chinese chive, leaves, sautéed; [6345] Carrot, regular (European type), root without skin, sautéed; [6346] Carrot, regular (European type), root without skin, deep-fried carrot; [6347] Carrot, regular (European type), skin, raw; [6348] Carrot, regular (European type), glazed carrot; [6353] Chayote, fruit, green-colored, raw; [6354] Broccoli, sprouts, raw; [6355] Spinach, leaves, summer harvest, raw; [6356] Spinach, leaves, winter harvest, raw; [6357] Spinach, leaves, summer harvest, boiled; [6358] Spinach, leaves, winter harvest, boiled; [6359] Spinach, leaves, all season, sautéed; [6360] Leaf green, "Mibuna", leaves, raw; [6361] Lettuce, head lettuce, crisp type, hydroculture, head, raw; [6362] Lettuce, Sang-chu lettuce, leaves, raw; [6363] Hosta grass, leaves, raw; [6364] Gourd, "Kanpyo" (dried gourd strips), simmered in a sweet and savory dashi-based broth; [6367] Japanese radishes, Daikon, root without skin, raw, grated; [6369] Japanese radishes, Daikon, root without skin, raw, grated, bleached in water; [6370] Tomatoes, fruit, dry; [6371] East Indian lotus root, rhizome, pickles, sweetened; [6372] Spinach, leaves, frozen, boiled; [6373] Spinach, leaves, frozen, sautéed; [8001] Mushrooms, winter mushrooms, raw; [8002] Mushrooms, winter mushrooms, boiled; [8003] Mushrooms, winter mushrooms, bottled in seasoning; [8004] Mushrooms, tree ears, Cloud ear fungus, dried, raw; [8005] Mushrooms, tree ears, Cloud ear fungus, dried, boiled; [8006] Mushrooms, tree ears, Cloud ear, dried, raw; [8007] Mushrooms, tree ears, Cloud ear, dried, boiled; [8008] Mushrooms, tree ears, white jelly fungus, dried, raw; [8009] Mushrooms, tree ears, white jelly fungus, dried, boiled; [8010] Mushrooms, abalone mushrooms, raw; [8013] Mushrooms, "Shiitake", dried, raw; [8014] Mushrooms, "Shiitake", dried, boiled; [8015] Mushrooms, "Hatakeshimeji", raw; [8016] Mushrooms, beech mushrooms, raw; [8017] Mushrooms, beech mushrooms, boiled; [8018] Mushrooms, "Honshimeji", raw; [8019] Mushrooms, golden oyster mushrooms, raw; [8020] Mushrooms, "Nameko", with harvesting the whole Nameko, raw; [8021] Mushrooms, "Nameko", with harvesting the whole Nameko, boiled; [8022] Mushrooms, "Nameko", canned in brine; [8024] Mushrooms, phoenix mushrooms, raw; [8025] Mushrooms, king oyster mushrooms, raw; [8026] Mushrooms, oyster mushrooms, raw; [8027] Mushrooms, oyster mushrooms, boiled; [8028] Mushrooms, "Maitake", raw; [8029] Mushrooms, "Maitake", boiled; [8030] Mushrooms, "Maitake", dried, raw; [8031] Mushrooms, button mushrooms, fresh, raw; [8032] Mushrooms, button mushrooms, fresh, boiled; [8033] Mushrooms, button mushrooms, canned in brine, solids; [8034] Mushrooms, "Matsutake", raw; [8037] Mushrooms, winter mushrooms, sautéed ; [8038] Mushrooms, tree ears, Cloud ear fungus, dried, rehydrated and sautéed; [8039] Mushrooms, "Shiitake", bed-log cultivation, fresh, raw; [8040] Mushrooms, "Shiitake", bed-log cultivation, fresh, boiled; [8041] Mushrooms, "Shiitake", bed-log cultivation, fresh, sautéed; [8042] Mushrooms, "Shiitake", wood-log cultivation, fresh, raw; [8043] Mushrooms, "Shiitake", wood-log cultivation, fresh, boiled; [8044] Mushrooms, "Shiitake", wood-log cultivation, fresh, sautéed; [8045] Mushrooms, "Hatakeshimeji", boiled; [8046] Mushrooms, beech mushrooms, sautéed; [8047] Mushrooms, "Honshimeji", boiled; [8048] Mushrooms, king oyster mushrooms, boiled; [8049] Mushrooms, king oyster mushrooms, baked; [8050] Mushrooms, king oyster mushrooms, sautéed; [8051] Mushrooms, "Maitake", sautéed; [8052] Mushrooms, button mushrooms, fresh, sautéed; [8053] Mushrooms, "Shiitake", dried, simmered in a sweet and savory dashi-based broth; [8054] Mushrooms, tree ears, Cloud ear fungus, fresh, raw; [9001] Algae, Sea lettuce, dried; [9002] Algae, green laver, dried; [9003] Algae, purple laver, dried; [9004] Algae, purple laver, dried, toasted; [9005] Algae, purple laver, dried, seasoned and toasted; [9006] Algae, "Arame", steamed and dried; [9007] Algae, "Iwa-nori", dried; [9009] Algae, "Ego-nori", "Okyuto" (algae jelly); [9010] Algae, "Ogo-nori", salted products, desalted; [9013] Algae, kombu, "Enaga-oni-kombu", dried; [9014] Algae, kombu, "Gagome-kombu", dried; [9015] Algae, kombu, "Naga-kombu", dried; [9016] Algae, kombu,

Table S2 (continued)

Variables	Food codes and food names[a]
	"Hosome-kombu", dried; [9017] Algae, kombu, "Ma-kombu", dried, raw; [9018] Algae, kombu, "Mitsuishi-kombu", dried; [9019] Algae, kombu, "Rishiri-kombu", dried; [9020] Algae, kombu, "Kizami-kombu" (dried and cut into thin strips); [9021] Algae, kombu, "Kezuri-kombu" (dried and thinly shaved); [9022] Algae, kombu, "Shio-kombu" (seasoned and dried) ; [9026] Algae, "Tengusa", "Tokoroten" (Gelidium jelly); [9027] Algae, "Tengusa", agar-agar; [9028] Algae, "Tengusa", agar jelly; [9029] Algae, "Tosaka-nori", red, salted products, desalted; [9030] Algae, "Tosaka-nori", green, salted products, desalted; [9032] Algae, "Hitoegusa", dried; [9034] Algae, "Fu-nori", dried; [9035] Algae, "Matsumo", dried; [9037] Algae, "Okinawa-mozuku", salted products, desalted; [9038] Algae, "Mozuku", salted products, desalted; [9039] Algae, "Wakame", raw; [9040] Algae, "Wakame", dried; [9041] Algae, "Wakame", dried, soaked in water; [9042] Algae, "Wakame", dried, "Ita-wakame" (made into sheets and dried); [9043] Algae, "Wakame", "Haiboshi" (coated with ash and dried), soaked in water; [9044] Algae, "Wakame", cut and dried; [9045] Algae, "Wakame", blanched and salted products, desalted, raw; [9046] Algae, "Wakame", stipe and center vein, blanched and salted products, desalted; [9047] Algae, "Wakame", fruit-bearing leaves, raw; [9049] Algae, "Tengusa", agar-agar powder; [9050] Algae, "Hijiki", boiled and dried, stainless steel pot process, raw; [9051] Algae, "Hijiki", boiled and dried, stainless steel pot process, rehydrated and boiled; [9052] Algae, "Hijiki", boiled and dried, stainless steel pot process, rehydrated and sautéed; [9053] Algae, "Hijiki", boiled and dried, steel pot process, raw; [9054] Algae, "Hijiki", boiled and dried, steel pot process, rehydrated and boiled; [9055] Algae, "Hijiki", boiled and dried, steel pot process, rehydrated and sautéed

[a] Food codes and food names were based on the eighth revised edition of the Standard Tables of Food Composition in Japan [46].
[b] The dishes containing each food code were listed and then the staple foods were identified by checking the name of the dish or menu (see Table 1). For the other variables, food intake was calculated by summing the listed food items.
[c] Mushrooms and seaweeds were also included in this category.

Table S3 Associations between meal context variables

	Day type		Eating location			Eating companion			Season			
	**Working or school days**	**Non-working or non-school days**	**At home**	**Restaurant**	**Other places**	**Alone**	**With one person**	**With two or more people**	**Spring**	**Summer**	**Fall**	**Winter**
**Meal type**												
Breakfast	10453 (52.6)	9437 (47.5)	18413 (92.6)	155 (0.8)	1322 (6.7)	10191 (51.2)	6456 (32.5)	3243 (16.3)	4980 (25.0)	4961 (24.9)	4987 (25.1)	4962 (25.0)
Lunch	11565 (53.8)	9923 (46.2)	9607 (44.7)	2671 (12.4)	9210 (42.9)	9587 (44.6)	5583 (26.0)	6318 (29.4)	5376 (25.0)	5381 (25.0)	5354 (24.9)	5377 (25.0)
Dinner	11682 (53.4)	10179 (46.6)	19711 (90.2)	1339 (6.1)	811 (3.7)	6286 (28.8)	8037 (36.8)	7538 (34.5)	5459 (25.0)	5455 (25.0)	5470 (25.0)	5477 (25.1)
**Day type**												
Working or school days	-	-	21442 (63.6)	1883 (5.6)	10375 (30.8)	15848 (47.0)	7955 (23.6)	9897 (29.4)	8553 (25.4)	8151 (24.2)	8599 (25.5)	8397 (24.9)
Non-working or non-school days	-	-	26289 (89.0)	2282 (7.7)	968 (3.3)	10216 (34.6)	12121 (41.0)	7202 (24.4)	7262 (24.6)	7646 (25.9)	7212 (24.4)	7419 (25.1)
**Eating location**												
At home	-	-	-	-	-	19290 (40.4)	17353 (36.4)	11088 (23.2)	11863 (24.9)	11973 (25.1)	11935 (25.0)	11960 (25.1)
Restaurant	-	-	-	-	-	1321 (31.7)	1572 (37.7)	1272 (30.5)	1076 (25.8)	1066 (25.6)	1004 (24.1)	1019 (24.5)
Other places	-	-	-	-	-	5453 (48.1)	1151 (10.2)	4739 (41.8)	2876 (25.4)	2758 (24.3)	2872 (25.3)	2837 (25.0)
**Eating companion**												
Alone	-	-	-	-	-	-	-	-	6605 (25.3)	6534 (25.1)	6480 (24.9)	6445 (24.7)
With one person	-	-	-	-	-	-	-	-	4994 (24.9)	5097 (25.4)	4944 (24.6)	5041 (25.1)
With two or more people	-	-	-	-	-	-	-	-	4216 (24.7)	4166 (24.4)	4387 (25.7)	4330 (25.3)

Values are shown as numbers (%). Significant associations between meal context variables were identified using Chi-square tests with a Bonferroni-adjusted significance level of $P < 0.005$, based on 10 comparisons (0.05/10). All the following variable pairs showed statistically significant associations: meal type and eating location, meal type and eating companion, day type and eating location, day type and eating companion, day type and season, and eating location and eating companion. Cells corresponding to these variable pairs are shaded in gray.

Table S4 Meal context and food types in relation to salt intake (g/meal) among 2443 plausible EI reporters[a]

	**Model 0** (Null model)	**Model 1** (Meal-level factors)	**Model 2** (Individual-level factors)	**Model 3** (Meal- and individual-level factors)
**Intercept**	3.48 (3.45, 3.52)***	3.49 (3.45, 3.52)***	3.48 (3.45, 3.51)***	3.49 (3.46, 3.51)***
**Meal context**				
Meal type (ref: breakfast)				
Lunch		0.50 (0.46, 0.54)***		0.50 (0.46, 0.54)***
Dinner		0.87 (0.82, 0.91)***		0.87 (0.82, 0.91)***
Day type (ref: working or school days)				
Non-working or non-school days		0.09 (0.05, 0.13)***		0.09 (0.05, 0.13)***
Eating location (ref: at home)				
Restaurant		0.40 (0.34, 0.46)***		0.40 (0.34, 0.46)***
Other places[b]		-0.19 (-0.23, -0.14)***		-0.19 (-0.23, -0.14)***
Eating companion (ref: alone)				
With one other person		0.08 (0.05, 0.12)***		0.08 (0.05, 0.12)***
With two or more people		-0.01 (-0.05, 0.03)		-0.01 (-0.05, 0.03)
Season (ref: spring)				
Summer		-0.05 (-0.09, -0.02)**		-0.05 (-0.09, -0.02)**
Fall		0.09 (0.05, 0.12)***		0.09 (0.05, 0.12)***
Winter		0.10 (0.07, 0.14)***		0.10 (0.07, 0.14)***
**Food type**				
Staple food (ref: no staple food)				
Rice		1.06 (1.03, 1.09)***		1.06 (1.03, 1.09)***
Bread		0.71 (0.67, 0.75)***		0.71 (0.67, 0.75)***
Noodles		-0.13 (-0.16, -0.10)***		-0.13 (-0.16, -0.10)***
Other staple foods		0.31 (0.19, 0.43)***		0.31 (0.19, 0.43)***
Other foods				
Soup (consumed; ref: not consumed)		0.13 (0.10, 0.16)***		0.13 (0.10, 0.16)***
Pickles (consumed; ref: not consumed)		0.29 (0.25, 0.34)***		0.29 (0.25, 0.34)***
Fruit (consumed; ref: not consumed)		0.57 (0.54, 0.60)***		0.57 (0.54, 0.60)***
Reduced-salt seasonings[c] (consumed, ref: not consumed)		0.57 (0.54, 0.61)***		0.57 (0.54, 0.61)***
Herbs and spices (consumed; ref: not consumed)		0.34 (0.30, 0.39)***		0.34 (0.30, 0.39)***
Citrus juice and vinegar (consumed; ref: not consumed)		0.35 (0.34, 0.35)***		0.35 (0.34, 0.35)***

Table S4 (continued)

	**Model 0** (Null model)	**Model 1** (Meal-level factors)	**Model 2** (Individual-level factors)	**Model 3** (Meal- and individual-level factors)
Moderately processed meat and seafood (consumed; ref: not consumed)		0.38 (0.36, 0.39)***		0.38 (0.36, 0.39)***
Highly processed meat and seafood (consumed; ref: not consumed)		0.13 (0.10, 0.16)***		0.13 (0.10, 0.16)***
Alcoholic beverages (consumed; ref: not consumed)		0.29 (0.25, 0.34)***		0.29 (0.25, 0.34)***
Salt-based seasonings[c] (continuous, unit[d]: 12.5 g/meal)		0.57 (0.54, 0.60)***		0.57 (0.54, 0.60)***
Vegetables[e] (continuous, unit[d]: 80.0 g/meal)		0.57 (0.54, 0.61)***		0.57 (0.54, 0.61)***
**Individual-level variables**				
Age (years)			0.003 (0.0003, 0.005)*	0.002 (-0.000004, 0.005)
Body mass index (kg/m^2)			0.01 (0.01, 0.02)***	0.02 (0.01, 0.02)***
Energy intake (kcal/day)			0.001 (0.001, 0.001)***	0.001 (0.001, 0.001)***
Sex (ref: male)				
Female			-0.15 (-0.23, -0.08)***	-0.16 (-0.24, -0.09)***
Living status (ref: living with others)				
Living alone			0.07 (-0.02, 0.15)	0.07 (-0.02, 0.16)
Education level (ref: junior high school or high school)				
Junior college or technical school			-0.01 (-0.08, 0.06)	-0.01 (-0.08, 0.06)
University or higher			-0.05 (-0.12, 0.02)	-0.05 (-0.12, 0.02)
Other			-0.15 (-0.50, 0.20)	-0.15 (-0.51, 0.20)
Employment status (ref: unemployed)				
Student			-0.09 (-0.27, 0.09)	-0.10 (-0.28, 0.09)
Part-time job			-0.04 (-0.14, 0.06)	-0.04 (-0.14, 0.06)
Full-time job			-0.06 (-0.14, 0.03)	-0.05 (-0.15, 0.04)
Annual household income[f] (ref: < 5 million Japanese yen)				
≥ 5 to < 8 million			-0.06 (-0.13, 0.01)	-0.06 (-0.13, 0.01)
≥ 8 million			-0.02 (-0.09, 0.06)	-0.02 (-0.09, 0.06)
Smoking status (ref: current smoker)				
Former smoker			-0.22 (-0.31, -0.13)***	-0.24 (-0.33, -0.14)***
Never smoker			-0.21 (-0.29, -0.13)***	-0.22 (-0.30, -0.14)***

Table S4 (continued)

	**Model 0** (Null model)	**Model 1** (Meal-level factors)	**Model 2** (Individual-level factors)	**Model 3** (Meal- and individual-level factors)
Residential area (ref: Hokkaido and Tohoku)				
Kanto			-0.04 (-0.13, 0.06)	-0.03 (-0.13, 0.06)
Hokuriku and Tokai			-0.15 (-0.26, -0.05)**	-0.16 (-0.26, -0.05)**
Kinki			-0.25 (-0.36, -0.14)***	-0.25 (-0.36, -0.14)***
Chugoku and Shikoku			-0.16 (-0.28, -0.05)**	-0.16 (-0.27, -0.04)**
Kyushu and Okinawa			-0.33 (-0.44, -0.21)***	-0.33 (-0.44, -0.21)***
Municipality type (ref: metropolis[g])				
Other cities			-0.01 (-0.07, 0.06)	-0.01 (-0.07, 0.06)
Towns and villages			0.01 (-0.09, 0.11)	0.004 (-0.10, 0.11)
Self-reported medical history[h] (ref: no)				
Yes			0.01 (-0.07, 0.08)	0.004 (-0.07, 0.08)
**Variance components (random effects)**				
Level 2 intercept	0.53***	0.65***	0.24***	0.36***
Residual	4.62***	2.21***	4.63***	2.21***
**Model Summary**				
Akaike information criterion	249810	210114	248663	208987
Intraclass correlation coefficient	0.10	0.23	0.05	0.14
Design effect	3.27	5.98	2.10	4.07

EI, energy intake; Ref, reference category.

[a] A total of 56,449 meals were analyzed. The dependent variable was absolute salt intake (g) per meal. Regression coefficients with 95% confidence intervals (in parentheses) are shown for meal context, food types, and individual characteristics. Other values are parameter estimates, with 95% confidence intervals indicated in parentheses if available. The regression coefficients represent the change in absolute salt intake at meals for a one-unit increase in salt-based seasonings, vegetables, age, body mass index, and energy intake. For other independent variables, the regression coefficients represent the difference in absolute salt intake at meals compared to the reference category. $^{*}P < 0.05$, $^{**}P < 0.01$, $^{***}P < 0.001$.

[b] Examples of other places include workplaces, schools, nursing-care facilities, parks, cars, and other people's houses.

[c] Except for those used in soups.

[d] The values were determined based on the median intake of each food item among consumers across all meals.

[e] Mushrooms and seaweeds were also included in this category.

[f] On June 10, 2025, 5 million Japanese yen was worth 34,507 US dollars, and 8 million yen was worth 55,212 US dollars.

[g] Government ordinance-designated cities and special wards of Tokyo.

[h] At least one of the following: hypertension, stroke, dyslipidemia, diabetes mellitus, hyperuricemia, liver disease, kidney disease, heart disease, and cancer.

Table S5 Meal context and food types in relation to salt intake density (g/100 kcal) among 2443 plausible EI reporters[a]

	**Model 0** (Null model)	**Model 1** (Meal-level factors)	**Model 2** (Individual-level factors)	**Model 3** (Meal- and individual-level factors)
**Intercept**	0.56 (0.56, 0.57)***	0.56 (0.56, 0.57)***	0.56 (0.56, 0.57)***	0.56 (0.56, 0.57)***
**Meal context**				
Meal type (ref: breakfast)				
Lunch		0.03 (0.02, 0.03)***		0.03 (0.02, 0.03)***
Dinner		0.01 (0.002, 0.02)*		0.01 (0.002, 0.02)*
Day type (ref: working or school days)				
Non-working or non-school days		0.01 (-0.001, 0.01)		0.006 (-0.001, 0.01)
Eating location (ref: at home)				
Restaurant		-0.01 (-0.02, 0.0004)		-0.01 (-0.02, 0.0004)
Other places[b]		-0.01 (-0.02, -0.01)**		-0.01 (-0.02, -0.006)**
Eating companion (ref: alone)				
With one other person		0.00 (-0.01, 0.01)		0.001 (-0.005, 0.008)
With two or more people		-0.01 (-0.02, -0.005)**		-0.01 (-0.02, -0.005)**
Season (ref: spring)				
Summer		-0.001 (-0.01, 0.01)		-0.001 (-0.007, 0.006)
Fall		0.01 (0.01, 0.02)***		0.01 (0.01, 0.02)***
Winter		0.01 (0.00, 0.02)***		0.01 (0.005, 0.02)***
**Food type**				
Staple food (ref: no staple food)				
Rice		-0.11 (-0.12, -0.10)***		-0.11 (-0.12, -0.10)***
Bread		-0.11 (-0.12, -0.10)***		-0.11 (-0.12, -0.10)***
Noodles		0.34 (0.33, 0.35)***		0.34 (0.33, 0.35)***
Other staple foods		-0.15 (-0.17, -0.14)***		-0.15 (-0.17, -0.14)***
Other foods				
Soup (consumed; ref: not consumed)		0.21 (0.20, 0.21)***		0.21 (0.20, 0.21)***
Pickles (consumed; ref: not consumed)		0.10 (0.09, 0.10)***		0.10 (0.09, 0.10)***
Fruit (consumed; ref: not consumed)		-0.09 (-0.10, -0.08)***		-0.09 (-0.10, -0.08)***
Reduced-salt seasonings[c] (consumed, ref: not consumed)		0.03 (0.01, 0.06)**		0.03 (0.01, 0.06)**
Herbs and spices (consumed; ref: not consumed)		-0.01 (-0.01, -0.001)*		-0.01 (-0.01, -0.001)*
Citrus juice and vinegar (consumed; ref: not consumed)		0.03 (0.03, 0.04)***		0.03 (0.03, 0.04)***

Table S5 (continued)

	Model 0 (Null model)	Model 1 (Meal-level factors)	Model 2 (Individual-level factors)	Model 3 (Meal- and individual-level factors)
Moderately processed meat and seafood (consumed; ref: not consumed)		0.06 (0.06, 0.07)***		0.06 (0.06, 0.07)***
Highly processed meat and seafood (consumed; ref: not consumed)		0.03 (0.03, 0.04)***		0.03 (0.03, 0.04)***
Alcoholic beverages (consumed; ref: not consumed)		-0.08 (-0.08, -0.07)***		-0.08 (-0.08, -0.07)***
Salt-based seasonings[c] (continuous, unit[d]: 12.5 g/meal)		0.03 (0.03, 0.03)***		0.03 (0.03, 0.03)***
Vegetables[e] (continuous, unit[d]: 80.0 g/meal)		0.02 (0.01, 0.02)***		0.02 (0.01, 0.02)***
**Individual-level variables**				
Age (years)			0.001 (0.001, 0.001)***	0.0009 (0.0005, 0.001)***
Body mass index (kg/m^2)			0.001 (-0.0003, 0.002)	0.001 (-0.0003, 0.002)
Energy intake (kcal/day)			0.0000 (-0.0001, -0.00003)***	-0.00004 (-0.00005, -0.00003)***
Sex (ref: male)				
Female			0.01 (-0.003, 0.02)	0.01 (-0.003, 0.02)
Living status (ref: living with others)				
Living alone			0.0004 (-0.01, 0.01)	0.0004 (-0.01, 0.01)
Education level (ref: junior high school or high school)				
Junior college or technical school			0.002 (-0.01, 0.01)	0.002 (-0.01, 0.01)
University or higher			-0.01 (-0.02, 0.01)	-0.01 (-0.02, 0.01)
Other			-0.01 (-0.07, 0.05)	-0.01 (-0.07, 0.05)
Employment status (ref: unemployed)				
Student			-0.004 (-0.03, 0.03)	-0.004 (-0.03, 0.03)
Part-time job			-0.02 (-0.04, -0.01)*	-0.02 (-0.04, -0.01)*
Full-time job			-0.03 (-0.05, -0.02)***	-0.03 (-0.05, -0.02)***
Annual household income[f] (ref: < 5 million Japanese yen)				
≥ 5 to < 8 million			-0.002 (-0.01, 0.01)	-0.002 (-0.01, 0.01)
≥ 8 million			0.001 (-0.01, 0.01)	0.001 (-0.01, 0.01)
Smoking status (ref: current smoker)				
Former smoker			-0.02 (-0.03, -0.01)**	-0.02 (-0.03, -0.005)**
Never smoker			-0.01 (-0.02, 0.002)	-0.01 (-0.02, 0.002)

Table S5 (continued)

	**Model 0** (Null model)	**Model 1** (Meal-level factors)	**Model 2** (Individual-level factors)	**Model 3** (Meal- and individual-level factors)
Residential area (ref: Hokkaido and Tohoku)				
Kanto			-0.02 (-0.03, -0.002)*	-0.02 (-0.03, -0.002)*
Hokuriku and Tokai			-0.03 (-0.04, -0.01)***	-0.03 (-0.04, -0.01)***
Kinki			-0.06 (-0.08, -0.05)***	-0.06 (-0.08, -0.04)***
Chugoku and Shikoku			-0.04 (-0.06, -0.02)***	-0.04 (-0.06, -0.02)***
Kyushu and Okinawa			-0.06 (-0.08, -0.04)***	-0.06 (-0.08, -0.04)***
Municipality type (ref: metropolis[g])				
Other cities			0.01 (-0.01, 0.02)	0.01 (-0.005, 0.02)
Towns and villages			0.02 (0.00, 0.03)*	0.02 (0.0006, 0.03)*
Self-reported medical history[h] (ref: no)				
Yes			0.005 (-0.01, 0.02)	0.005 (-0.007, 0.02)
**Variance components (random effects)**				
Level 2 intercept	0.01***	0.01***	0.01	
Residual	0.11***	0.07***	0.11	0.01***
**Model Summary**				
Akaike information criterion	38557	15659	38332	
Intraclass correlation coefficient	0.07	0.12	0.06	38332
Design effect	2.52	3.64	2.28	0.06

EI, energy intake; Ref, reference category.

[a] A total of 56,449 meals were analyzed. The dependent variable was salt intake density (g/100 kcal) per meal. Regression coefficients with 95% confidence intervals (in parentheses) are shown for meal context, food types, and individual characteristics. Other values are parameter estimates, with 95% confidence intervals indicated in parentheses if available. The regression coefficients represent the change in salt intake at meals for a one-unit increase in salt-based seasonings, vegetables, age, body mass index, and energy intake. For other independent variables, the regression coefficients represent the difference in salt intake at meals compared to the reference category. $^{*}P < 0.05$, $^{**}P < 0.01$, $^{***}P < 0.001$.

[b] Examples of other places include workplaces, schools, nursing-care facilities, parks, cars, and other people's houses.

[c] Except for those used in soups.

[d] The values were determined based on the median intake of each food item among consumers across all meals.

[e] Mushrooms and seaweeds were also included in this category.

[f] On June 10, 2025, 5 million Japanese yen was worth 34,507 US dollars, and 8 million yen was worth 55,212 US dollars.

[g] Government ordinance-designated cities and special wards of Tokyo.

[h] At least one of the following: hypertension, stroke, dyslipidemia, diabetes mellitus, hyperuricemia, liver disease, kidney disease, heart disease, and cancer.
